# Supplementary material for: Identification of barriers, facilitators and system-based implementation strategies to increase teleophthalmology use for diabetic eye screening in a rural US primary care clinic: a qualitative study
Source: BMJ Open. 2019 Feb 18;9(2):e022594. doi: 10.1136/bmjopen-2018-022594 (PMC6398662; doi:10.1136/bmjopen-2018-022594)
Supplement: Supplementary file 1 [file bmjopen-2018-022594supp001.pdf]

Supplemental Appendix S1. Patient Interview Guide

Introduction:

Thank you so much for taking the time to meet with me. Our research team at the University of Wisconsin is working with the Mile Bluff Medical Center to learn more about patient experiences with diabetic eye exams.

You were invited to participate because you are a Mile Bluff Medical Center patient who has diabetes. I expect our conversation will last about 45 minutes. At the end of our discussion, I have a few questions about your background. Participation in this interview is voluntary. You can stop the interview at any time and if there are any questions you don't want to answer you can just tell me to skip those. Everything you tell me today will be kept confidential. Only our research team led by Dr. Yao Liu, a UW eye doctor, will have access to this information.

I will be audio recording this interview so that I can review our discussion later and make sure I accurately get all the ideas and opinions that you share. This interview is about <u>your</u> personal experience with eye exams. There are no "right" or "wrong" answers.

Do you have any questions before we begin? If you think of any questions as we go along, feel free to ask them any time.

Part I. Interview Questions

First, I'd like to ask you some general questions about how you take care of your diabetes.

What are things you do each day that are related to your diabetes?

Prompts:

- ☐ There are many things people do to take care of their diabetes. What do you see as the biggest priorities for you?
- ☐ *[if none]* What are some of the things your doctor may have discussed with you that may be important for people with diabetes to do?
- ☐ Are there things that you watch out for that tell you your diabetes may be getting worse?

Now I'd like to show you some pictures. These photos show two ways of doing eye checks for people with diabetes. The top photo shows a Traditional Eye Exam, where an eye doctor uses eye drops to dilate and examine your eyes. The bottom photo shows an Eye Photo Test, where a technician uses a special camera to take photos of your eyes.

Some people get yearly eye checks as part of their diabetes care. What do you think are the advantages of this? What are possible disadvantages to getting these eye checks?

Prompts:

- ☐ How do you know when it is time for you to get an eye check for diabetes?
- ☐ Why did you choose to get your eyes checked?

In your experience, how easy is it to regularly get diabetic eye checks as compared to *[use their example about something they do for their diabetes]*?

Prompts:

- ☐ Can you tell me more about why you find it easier/harder?

- ☐ You mentioned *[x]* as one thing that makes it hard to regularly get eye exams. Can you tell me more about that? What else might make it hard?
- ☐ What do you think might make it easier for you or other people with diabetes in your community to regularly get eye exams?

How do you find information about diabetic eye checks?

Prompts:

- ☐ From where do you get this information?
- ☐ Are there helpful resources here in your community?
- ☐ What support or resources do you think would help people learn more about this or help them get their eyes checked?
- ☐ *[If they don't currently get information about eye exams.]* Where do you get information about what you need to do related to diabetes?

[If patient has not had teleophthalmology, jump to page 4: Version II. Clinical Eye Exam]

Version I. Teleophthalmology

I believe you had the Eye Photo Test *[point to photo]* ***[x]*** **weeks/months ago, is this correct?**

Tell me about your experience starting with how you first learned about the Eye Photo Test.

Prompts:

- ☐ Do you remember who explained this test to you?
- ☐ What did they tell you about it?
- ☐ Did you get all the information you wanted?
- ☐ What made you decide to have the Eye Photo Test?
- ☐ Why do you think your doctor referred you for the Eye Photo Test?

Next, I have a few questions about scheduling the Eye Photo Test and getting to your appointment.

How easy was it to schedule the Eye Photo Test?

Prompts:

- ☐ How long did you wait between the time you scheduled the Eye Photo Test and when you had the appointment? (e.g. a few days, weeks or months)
- ☐ How easy was it to get to the clinic for the photo eye test? Did you drive yourself or did someone else drive you? How long did it take to get from your home to the clinic?

Now I'd like you to tell me about your experience with the Eye Photo Test itself.

Prompts:

- ☐ About how long did the Eye Photo Test take?
- ☐ Did you receive results from your Eye Photo Test?
- ☐ *[If YES]* How quickly did you get those results? Is there anything that could be changed or improved about the way you receive the results?
- ☐ *[If NO]* Did you want to receive the results from the Eye Photo Test?
- ☐ How would you like to receive your Eye Photo Test results? (e.g. letter in the mail or phone call)

How would you explain the Eye Photo Test to a friend or family member?

Prompts:

- ☐ Would you be willing to take the Eye Photo Test again? Why or why not?

Do you think that there is anything that could be improved about your experience with the Eye Photo Test?

Prompts:

- □ How can we make the Eye Photo Test more available to other people in your community who have diabetes?
- □ For your community, do you think $20 is a reasonable cost for the Eye Photo Test?

Do you plan to have another diabetic eye check in the future?

Prompts:

- □ *[If YES]* How soon and where might you schedule this? [verify what type of exam – Traditional Eye Exam vs Eye Photo Test]
- □ *[If NO]* Why not?

Let's look at the picture of the Traditional Eye Exam *[point to photo]***. Have you ever had this exam where an eye doctor uses eye drops to dilate and examine your eyes?**

Prompts:

- □ *[If YES]* Tell me about your experience with this exam. What was it like?

Does your insurance cover Traditional Eye Exams? How much do you normally pay to have a Traditional Eye Exam?

Let's look at the picture of the two types of eye checks again. If you had to choose between having your eyes checked using a Traditional Eye Exam or an Eye Photo Test, which would you prefer? Why?

[Jump to Part II on page 5]

Version II. Clinical Eye Exam

I believe you have not had the Eye Photo Test *[point to photo]***, is this correct?**

Now, let's look at the picture of the Traditional Eye Exam *[point to photo]***. Have you ever had this exam where an eye doctor uses eye drops to dilate and examine your eyes? If so, do you recall roughly the last time you had a Traditional Eye Exam?**

Next, I have a few questions about scheduling the Traditional Eye Exam and getting to your appointment.

How easy was it to schedule the Traditional Eye Exam?

Prompts:

- □ How long did you wait between the time you scheduled the exam and when you had the appointment? (e.g. a few days, weeks or months)
- □ How easy was it to get to the clinic for the Traditional Eye Exam? Did you drive yourself or did someone else drive you? How long did it take to get from your home to the clinic?

Now I'd like you to tell me about your experience with the Traditional Eye Exam itself.

Prompts:

- □ About how long did the exam take?
- □ Did you receive results from your Traditional Eye Exam?
- □ *[If YES]* How quickly did you get those results? Is there anything that could be changed or improved about the way you receive the results?
- □ *[If NO]* How would you want to receive the results from the exam?

How would you describe the Traditional Eye Exam to a friend or family member?

Prompts:

- □ Would you be willing to have a Traditional Eye Exam again? Why or why not?

Do you plan to have another diabetic eye check in the future?

Prompts:

- □ *[If YES]* How soon and where might you schedule this? [verify what type of exam – Traditional Eye Exam vs Eye Photo Test]
- □ *[If NO]* Why not?

Does your insurance cover Traditional Eye Exams? How much do you normally pay to have a Traditional Eye Exam?

Let's look at the picture of the two types of eye exams again. If you had to choose between having your eyes examined using a Traditional Eye Exam or an Eye Photo Test, which would you prefer? Why?

Part II. Wrap-up and Demographics

Is there anything else you think is important for me to know about your experience with getting diabetic eye checks?

Thanks so much for all this great information. I really appreciate your sharing your experience with me.

As I mentioned earlier, there is just one last thing—a short list of questions to get some background information. Please understand that we only want this this information so that we have an accurate picture of who is involved in our study; we know the community is diverse and we want to be sure we hear many different perspectives and hear from people with a wide range of backgrounds. We are not making any judgments or assumptions about you based on this information.

I will read the questions to you or if you prefer, you can read the questions on your own.

[Read or hand patient page 6 to complete]

Patient Background Information

1. How long have you had diabetes?

a) Less than 5 years
b) Between 5-10 years
c) Between 10-15 years
d) More than 15 years

2. Do you drive yourself to your eye appointments? (If no, who drives you)?

a) Yes
b) No, my ______________________________ drives me to appointments

3. During regular clinic hours, are you easily able to get your eye appointments?

a) Yes
b) No

4. What level of school have you completed?

____

5. We know that health information is often written in a way that is complicated and hard to understand. How often do you need to have someone help you when you read instructions, pamphlets, or other written material from your doctor or pharmacy?

a) Never
b) Rarely
c) Sometimes
d) Often
e) Always
