# Supplementary material for: Identification of barriers, facilitators and system-based implementation strategies to increase teleophthalmology use for diabetic eye screening in a rural US primary care clinic: a qualitative study
Source: BMJ Open. 2019 Feb 18;9(2):e022594. doi: 10.1136/bmjopen-2018-022594 (PMC6398662; doi:10.1136/bmjopen-2018-022594)
Supplement: Supplementary file 2 [file bmjopen-2018-022594supp002.pdf]

Supplemental Appendix S2. Primary Care Provider (PCP) Interview Guide

Introduction:

Thank you so much for taking the time to talk with me. I'm going to start off by giving you an introduction and our goals for this interview.

As you know, in collaboration with Mile Bluff Medical Center (MBMC), our research team at the University of Wisconsin is studying primary care provider perspectives on diabetic eye exams.

You are being invited to participate in this interview because we are interested in learning more about your experience, as a primary care provider, with teleophthalmology and your ideas on ways to make it easier for you to use. We are also conducting separate interviews with patients to understand their experiences as well.

Participation in this study is voluntary. You may discontinue your participation at any time. There won't be any identifying information linked with your responses. Only researchers on this project will have access to the data gathered.

I will audiotape this interview so that I can take fewer notes as we talk and review our conversation later to ensure that I don't miss any information. The interview should take about 15-20 minutes. Please feel free to stop me at any time with questions or concerns.

Do you have any questions about the study before we begin?

Version I. Teleophthalmology

[NOTE: If PCP has not referred patients for teleophthalmology, skip to page 3: Version II. Clinical eye exam]

Tell me about your experience with teleophthalmology.

Prompts:

- ☐ Why did you choose to refer patients for teleophthalmology?
- ☐ What helped in that process? What didn't help in that process?
- ☐ Can you describe an instance when a referral did not happen and why?

What makes referring patients for diabetic eye exams easier or more difficult than other health maintenance screenings (e.g. mammography and colonoscopy)?

Now I'd like to ask you to walk me through your decision to refer a typical patient for teleophthalmology.

Prompts:

- ☐ Do you refer the patient? Do you have help from medical assistants or schedulers?
- ☐ How do you decide whether to refer a patient for teleophthalmology versus a traditional diabetic eye exam?
- ☐ What patient factors, such as their characteristics or preferences, influence your decision?
- ☐ What about non-patient factors that might influence your decision, such as how busy you are in clinic?
- ☐ When you see a patient with multiple health issues, at what point during a typical clinic visit might you bring up the topic of diabetic eye screening?

How do you explain teleophthalmology to the patient?

Prompts:

- □ How do patients typically respond?
- □ What makes you feel confident, or not, that the patient will obtain teleophthalmology screening?
- □ Of your patients that have had this test done, what kind of feedback do they give you?

Is there anything that might make easier for you to have your patients use teleophthalmology?

Prompts:

- □ Some examples may include:
  Interventions to identify patients eligible for eye screening
  Delegation of referrals to support staff
  Walk-in/flexible scheduling of eye screening
- □ Do you have any concerns or questions about this technology?
- □ Are there incentives or processes in place that encourage referring patients for this or other types of screening?

This has been very helpful. Before we conclude our conversation, is there anything else I should be asking you about teleophthalmology or diabetic eye exams that is important for me to know?

Thank you very much for your participation!

Version II. Clinical eye exam *[if PCP has not referred patients for teleophthalmology]*

Tell me about your experience with teleophthalmology.

Prompts:

- ☐ Do you have any concerns or questions about this technology?
- ☐ Have you considered having patients get teleophthalmology screening instead of a clinical diabetic eye exam? Why or why not?

Is there anything that might make it more likely for you to have your patients use teleophthalmology?

Prompts:

- ☐ For example:
  Interventions to identify patients eligible for eye screening
  Delegation of referrals to support staff
  Walk-in/flexible scheduling of eye screening
- ☐ Are there incentives or processes in place that encourage you or your colleagues to refer patients for this or other types of screening?

What makes referring patients for diabetic eye exams easier or more difficult than other health maintenance screenings (e.g. mammography and colonoscopy)?

Now I'd like you to walk me through the steps you or your staff take when helping patients get diabetic eye exams.

Prompts:

- ☐ Do you refer the patient? Do you have help from medical assistants or schedulers? Do patients refer themselves?
- ☐ How do you decide whether to refer a patient for a diabetic eye exam?
- ☐ What patient factors, such as their characteristics or preferences, influence your decision?
- ☐ What about non-patient factors that might influence your decision, such as how busy you are in clinic?
- ☐ When you see a patient with multiple health issues, at what point during a typical clinic visit do you bring up the topic of diabetic eye screening?
- ☐ Is there anything that could be improved about this process?

How do you explain diabetic eye exams to patients?

Prompts:

- ☐ How do patients typically respond?
- ☐ What makes you feel confident, or not, that the patient will obtain the exam?
- ☐ Of your patients that have had a diabetic eye exam, what kind of feedback do they give you?

This has been very helpful. Before we conclude our conversation, is there anything else I should be asking you about teleophthalmology or diabetic eye exams that is important for me to know?

Thank you very much for your participation!
