# Supplementary material for: Identification of barriers, facilitators and system-based implementation strategies to increase teleophthalmology use for diabetic eye screening in a rural US primary care clinic: a qualitative study
Source: BMJ Open. 2019 Feb 18;9(2):e022594. doi: 10.1136/bmjopen-2018-022594 (PMC6398662; doi:10.1136/bmjopen-2018-022594)
Supplement: Author's manuscript [file bmjopen-2018-022594.draft_revisions.pdf]

BMJ Open

BMJ Open is committed to open peer review. As part of this commitment we make the peer review history of every article we publish publicly available.

When an article is published we post the peer reviewers' comments and the authors' responses online. We also post the versions of the paper that were used during peer review. These are the versions that the peer review comments apply to.

The versions of the paper that follow are the versions that were submitted during the peer review process. They are not the versions of record or the final published versions. They should not be cited or distributed as the published version of this manuscript.

BMJ Open is an open access journal and the full, final, typeset and author-corrected version of record of the manuscript is available on our site with no access controls, subscription charges or pay-per-view fees (http://bmjopen.bmj.com).

If you have any questions on BMJ Open's open peer review process please email info.bmjopen@bmj.com

BMJ Open

System-based Implementation Strategies to Increase Teleophthalmology Use for Diabetic Eye Screening in Multi-Payer Primary Care Clinics: A Qualitative Study

Journal:	*BMJ Open*
Manuscript ID	bmjopen-2018-022594
Article Type:	Research
Date Submitted by the Author:	03-Mar-2018
Complete List of Authors:	Liu, Yao; University of Wisconsin-Madison School of Medicine and Public Health, Ophthalmology and Visual Sciences; University of Wisconsin-Madison School of Medicine and Public Health, Health Innovation Program<br>Zupan, Nicholas; University of Wisconsin-Madison School of Medicine and Public Health, Ophthalmology and Visual Sciences<br>Swearingen, Rebecca; University of Wisconsin-Madison School of Medicine and Public Health, Ophthalmology and Visual Sciences<br>Jacobson , Nora; University of Wisconsin Madison Institute for Clinical and Translational Research<br>Carlson, Julia; University of Wisconsin-Madison School of Medicine and Public Health, Ophthalmology and Visual Sciences<br>Mahoney, Jane E. ; University of Wisconsin School of Medicine and Public Health, Medicine<br>Klein, Ronald; University of Wisconsin-Madison School of Medicine and Public Health, Ophthalmology and Visual Sciences<br>Bjelland, Timothy; Mile Bluff Medical Center<br>Smith, Maureen; University of Wisconsin School of Medicine and Public Health, Health Innovation Program; University of Wisconsin School of Medicine and Public Health, Population Health Sciences, Family Medicine and Community Health
Keywords:	Diabetic retinopathy < DIABETES & ENDOCRINOLOGY, Organisation of health services < HEALTH SERVICES ADMINISTRATION & MANAGEMENT, PUBLIC HEALTH, Telemedicine < BIOTECHNOLOGY & BIOINFORMATICS, QUALITATIVE RESEARCH

SCHOLARONE™ Manuscripts

Title: System-based Implementation Strategies to Increase Teleophthalmology Use for Diabetic Eye Screening in Multi-Payer Primary Care Clinics: A Qualitative Study

Running Title: System-based Strategies for Teleophthalmology

Authors: Liu, Yao M.D.[1,2*]; Zupan, Nicholas J. M.P.H.[1]; Swearingen, Rebecca B.S.[1,2]; Jacobson, Nora A. Ph.D.[3]; Carlson, Julia N. B.S.[1]; Mahoney, Jane E. M.D.[4]; Klein, Ronald M.D. M.P.H.[1]; Bjelland, Timothy D. D.O.;[5] Smith, Maureen A. M.D. M.P.H. Ph.D.[2,6]

Affiliations:

[1]University of Wisconsin School of Medicine and Public Health, Department of Ophthalmology and Visual Sciences, Madison, Wisconsin, United States of America

[2]University of Wisconsin School of Medicine and Public Health, Health Innovation Program, Madison, Wisconsin, United States of America

[3]University of Wisconsin School of Medicine and Public Health, Institute for Clinical and Translational Research, Madison, Wisconsin, United States of America

[4]University of Wisconsin School of Medicine and Public Health, Department of Medicine, Madison, Wisconsin, United States of America

[5]Mile Bluff Medical Center, Mauston, Wisconsin, United States of America

[6]University of Wisconsin School of Medicine and Public Health, Departments of Population Health Sciences, Family Medicine and Community Health, Madison, Wisconsin, United States of America

For peer review only

***Corresponding Author:**

Yao Liu, MD

Assistant Professor, Department of Ophthalmology and Visual Sciences

University of Wisconsin School of Medicine and Public Health

2870 University Ave, Ste. 206

Madison, WI 53705

Phone: 608-263-1481

Fax: 608-263-7694

Email: liu463@wisc.edu

Word Count: 3837

Total Number of Tables and Figures: 4

Keywords: diabetic retinopathy, organization of health services, telemedicine, public health, qualitative research

For peer review only

For peer review only - http://bmjopen.bmj.com/site/about/guidelines.xhtml

ABSTRACT

Objective

Teleophthalmology for diabetic eye screening is an evidence-based intervention substantially underutilized in U.S multi-payer primary care clinics, even when equipment and trained personnel are readily available. We sought to identify patient and primary care provider (PCP) barriers, facilitators, as well as strategies to increase teleophthalmology use.

Design, Setting and Participants

We performed semi-structured individual interviews with 29 participants (20 patients with diabetes and 9 PCPs) in a rural, multi-payer primary care clinic following the establishment of a teleophthalmology program for diabetic eye screening. Both inductive and directed content analysis of interview transcripts were performed to identify barriers and facilitators to teleophthalmology use. The *Chronic Care Model* was used as a framework for development of the interview guide and for categorizing implementation strategies to increase teleophthalmology use.

Results

Major patient barriers to teleophthalmology use included being unfamiliar with teleophthalmology, misconceptions about diabetic eye screening, and logistical challenges. Major patient facilitators included a recommendation from the patient's PCP and factors related to convenience. Major PCP barriers to referring patients for teleophthalmology included difficulty identifying when patients are due for diabetic eye screening and being unfamiliar with

For peer review only - http://bmjopen.bmj.com/site/about/guidelines.xhtml

For peer review only

teleophthalmology. Major PCP facilitators included the ease of the referral process and communication of screening results. Based on our results, we developed a model that maps where these key patient and PCP barriers occur in the teleophthalmology referral process. Patients and PCPs also identified implementation strategies to directly address barriers and facilitators to teleophthalmology use.

Conclusions

Patients and PCPs have limited familiarity with teleophthalmology for diabetic eye screening. PCPs were expected to initiate teleophthalmology referrals, but reported significant difficulty identifying when patients are due for diabetic eye screening. System-based implementation strategies primarily targeting PCP barriers in conjunction with improved patient and provider education may increase teleophthalmology use in rural, multi-payer primary care clinics.

For peer review only

For peer review only - http://bmjopen.bmj.com/site/about/guidelines.xhtml

Article Summary

Strengths and limitations of this study:

- We used qualitative methods to capture the real-world perspectives of patients and providers regarding barriers and facilitators to teleophthalmology use in a multi-payer primary care clinic with an active teleophthalmology program.
- We identified and categorized implementation strategies directly suggested by patients and providers using the Chronic Care Model.
- All patients were Caucasian, native English speakers.
- Most patients in this study self-reported high levels of health literacy (85%), which was greater than that reported by rural adults from a similar population (70.9%).

Funding statement

This work was supported by NIH/NEI K23 EY026518-02 and a Wisconsin Partnership Program New Investigator Award. It was also supported, in part, by an institutional grant from Research to Prevent Blindness, Inc., New York, NY, USA to the University of Wisconsin School of Medicine and Public Health, Dept. of Ophthalmology and Visual Sciences. Additional support came from the Clinical and Translational Science Award (CTSA) program, through the NIH National Center for Advancing Translational Sciences (NCATS), grant UL1TR000427. Further support was received from the University of Wisconsin (UW) School of Medicine and Public Health, UW Health Innovation Program, the Wisconsin Partnership Program, and the UW Institute for Clinical and Translational Research-Community Academic Partnerships (ICTR-CAP) core.

For peer review only - http://bmjopen.bmj.com/site/about/guidelines.xhtml

For peer review only

The study sponsors had no role in the design and conduct of the study; collection, management, analysis, and interpretation of the data; preparation, review, or approval of the manuscript; and the decision to submit the manuscript for publication. The content is solely the responsibility of the authors and does not necessarily represent the official views of the NIH.

Competing interests statement: The authors have no conflicts of interest to disclose relevant to the subject matter in this manuscript.

Data sharing statement: The data set from which this study is based is available on request.

For peer review only

For peer review only - http://bmjopen.bmj.com/site/about/guidelines.xhtml

INTRODUCTION

Diabetic retinopathy affects an estimated 4.2 million Americans and is the most common cause of blindness in working-age U.S. adults.[1-2] Early diagnosis and treatment decrease the risk of severe vision loss by 90%, but fewer than half of the 29.1 million Americans with diabetes receive yearly retinopathy screening in accordance with recommended guidelines.[3-5] Teleophthalmology is an evidence-based intervention proven to substantially improve diabetic eye screening rates and reduce blindness from diabetes.[6] A retinal camera is used to image patients' eyes in a convenient location, such as a primary care clinic (where more than 90% of patients with diabetes obtain their care).[7] These images are then electronically-transmitted to and evaluated by specialists at a distant site. Patients needing additional eye care are then rapidly identified for further treatment. The prevalence of diabetes, and the demand for eye screening, is projected to double by 2050 without a concurrent increase in the supply of eye care providers.[8] Thus, there is an urgent need to expand teleophthalmology use to improve screening rates and respond to growing demand.

In England, the National Health Service achieves screening rates of over 80% using teleophthalmology and subsequently, diabetic retinopathy is no longer the leading cause of blindness in working-age English adults.[8-9] Presently, successful implementation of teleophthalmology in the U.S. is largely limited to single-payer or highly specialized health systems.[6,10-11] Teleophthalmology programs in these settings have achieved sustained screening rates as high as 80% or more. Success in multi-payer settings has been much more limited. A recent 5-year randomized-controlled trial in a multi-payer health system compared teleophthalmology to traditional screening methods (i.e. in-person dilated eye exams) and found initial improvement in screening rates with teleophthalmology, but screening rates declined

For peer review only - http://bmjopen.bmj.com/site/about/guidelines.xhtml

within 18 months and did not exceed 55% even when teleophthalmology became available to both groups.[12] However, this study did not utilize a systematic implementation approach designed to sustain integration of this technology into the primary care workflow.

Teleophthalmology is particularly well suited to rural populations, which have less access and greater travel distances to obtain eye care.[13-17] Rural communities are largely served by multi-payer health systems, which are less likely to encourage preventive services.[18] Unfortunately, studies show that simply providing access to teleophthalmology in multi-payer health systems is insufficient to either achieve or sustain initial improvements in screening rates.[11-12,18-20] Patient and provider-related barriers to teleophthalmology have been postulated, but are poorly understood.[12] These barriers may be magnified in rural populations because they older, poorer, less insured, less likely to receive guideline-concordant care, and experience more chronic diseases than those in urban areas.[13-15]

We hypothesize that while teleophthalmology addresses some logistical barriers to diabetic eye screening, it may not address other important patient and provider barriers in multi-payer health systems. Qualitative research methods are used in health services research to explore complex phenomena needing further explanatory analysis, such as real-world patient and provider barriers to teleophthalmology use, through a rich description of key perspectives.[21] We performed individual interviews to understand what prevents or motivates patients and primary care providers (PCPs) to use teleophthalmology, as well as strategies to increase teleophthalmology use, in a rural multi-payer health system with an existing teleophthalmology program.

RESEARCH DESIGN AND METHODS

For peer review only - http://bmjopen.bmj.com/site/about/guidelines.xhtml

Research Setting

We performed semi-structured interviews with patients with diabetes and PCPs at Mile Bluff Medical Center. Mile Bluff is a rural, multi-payer health system in Mauston, Wisconsin. A teleophthalmology program was established in 2015 in partnership with the University of Wisconsin-Madison. This program allows PCPs to refer patients for teleophthalmology with walk-in scheduling. Referrals are completed by PCPs in the Mile Bluff electronic health record (EHR). Retinal images are electronically-transmitted to and evaluated by University eye specialists. Imaging reports are then sent back to the PCP and patient within 1 week. Patients are charged $20 for the service and referred to local eye doctors for further care if found to have visually-significant eye disease. This teleophthalmology program was developed based on the 2011 American Telemedicine Association Telehealth Practice Recommendations for Diabetic Retinopathy.[22] In this setting, we deliberately chose not to tailor an implementation program to increase adoption or sustain use of teleophthalmology in order to allow assessment of the "natural history" of teleophthalmology use and establish baseline utilization from which to measure the effects of a future implementation program.

Interviews

We developed our semi-structured interview guides using the *Chronic Care Model,* a practical framework for improving chronic disease management and guideline-concordant diabetes care.[23-24] Following a literature search on barriers and facilitators to teleophthalmology for diabetic eye screening, the patient interview guide (Supplemental Appendix S1) was tested and further refined with input from the University of Wisconsin Community Advisors on Research Design and Strategies (CARDS®), a group of lay community

For peer review only - http://bmjopen.bmj.com/site/about/guidelines.xhtml

members trained to review patient research materials. The PCP interview guide (Supplemental Appendix S2) was tested and further refined with the input of PCPs from the University of Wisconsin Primary Care Academics to Transform Healthcare (PATH). Members of the University of Wisconsin Institute for Clinical and Translational Research-Community Academic Partnership (UW ICTR-CAP) Qualitative Research Group also reviewed and provided feedback on both interview guides.

Semi-structured, individual interviews to understand patient and PCP perspectives on teleophthalmology and diabetic eye screening were performed between July 2016 and April 2017 (1-2 years after the teleophthalmology program was established). All interviews were conducted by a research specialist (R.S.) with training in qualitative research and certification as a nursing assistant. Patient interviews were performed in-person (30-45 minutes) at a local library. PCP interviews were conducted over the phone (15-30 minutes) to accommodate their busy clinic schedules. Patients received $30 and PCPs received $50 compensation for their time. Interviews were audiotaped and transcribed verbatim with all personal identifiers redacted.

Study Sample

The sample of 20 adult patients with diabetes (with and without experience with teleophthalmology) and 9 PCPs was drawn from patients and providers at Mile Bluff Medical Center. Adult patients (18 year or older) with a diagnosis of diabetes were recruited who either: (1) had teleophthalmology imaging within the preceding 2 months or (2) expressed interest in participating in a research study when previously contacted in a quality improvement telephone survey on diabetic eye screening. All patients had a PCP from Mile Bluff Family or Internal Medicine. PCPs were recruited during a provider staff meeting with purposeful recruitment of

For peer review only - http://bmjopen.bmj.com/site/about/guidelines.xhtml

For peer review only

providers having a range of training backgrounds reflective of their representation at Mile Bluff. Sample sizes for both patients and PCPs were sufficient to reach informational redundancy in which no new information was obtained from additional interviews.[25]

Data Analysis

We began analyzing the interview transcripts using inductive analysis. Open coding of the first 5 transcripts was performed independently by 3 members of the research team (R.S.-research specialist and certified nursing assistant, N.Z.-research specialist with a Master of Public Health Degree, and Y.L.-clinical ophthalmologist and principal investigator). These research team members then met together with N.J.-Ph.D. qualitative methodologist to review these codes and agree upon a coding framework. A second-coding cycle was then performed by 1 research team member (N.Z.) to fit codes into higher-order categories. Consistency was ensured by dual-coding of every 5th transcript by the principal investigator (Y.L.). Codes were iteratively reviewed and further refined by the entire research team. Additionally, implementation strategies to increase teleophthalmology use were deductively coded and categorized using the *Chronic Care Model* as a framework for directed content analysis. The interview data and coding methods were also reviewed by members of the UW ICTR-CAP Qualitative Research Group. Data management was facilitated using NVivo software, Version 11.4.1 (QSR International, Melbourne, Australia).

Member checking to validate our data analysis was performed with a subset of interview participants (n=9 patients, n=6 PCPs) at two separate patient and provider stakeholder group meetings organized as part of a quality improvement initiative to increase diabetic eye screening at Mile Bluff.[26] Participating patients and providers judged our interpretation of the interview

For peer review only - http://bmjopen.bmj.com/site/about/guidelines.xhtml

data to be accurate and complete. Furthermore, the most important or "top" barriers and facilitators were identified through the Nominal Group Technique, in which each participant sequentially shares one additional idea with the group (until no new ideas are generated) and then all participants anonymously cast their written votes.[27] We followed the Standards for Reporting Qualitative Research (SRQR) checklist in our report of this study.[28]

Patient Involvement

Patients from the University of Wisconsin Community Advisors on Research Design and Strategies (CARDS®) were involved in the design of the study by providing feedback on development of the patient interview guide. In addition, a patient stakeholder group (Mile Bluff Diabetes Patient Advisory Council) was established among participants in this study to provide member-checking of our results and advise on dissemination of the results.

Ethics/Institutional Review Board Approval

The University of Wisconsin School of Medicine and Public Health Human Subjects Institutional Review Board determined that this interview research met criteria for exemption. Verbal consent was obtained from all study participants.

RESULTS

Patient and Provider Characteristics

All patients were Caucasian adults diagnosed with type-2 diabetes (Table 1). Fifty percent of patients had experience with teleophthalmology and most (85%) self-reported high health literacy in response to the Single Item Literacy Screener.[29] PCPs came from a variety of

For peer review only - http://bmjopen.bmj.com/site/about/guidelines.xhtml

For peer review only

training backgrounds and were predominantly male (77.8%) (Table 1). Most had been in practice for over 10 years (77.8%) and had referred patients for teleophthalmology (87.5%).

Table 1. Patient and Primary Care Provider Demographics

Participant Characteristics	Median or Percentage
Patients (n=20)	
Age	67 years (range: 46-86 years)
Male	55%
Ethnicity (self-reported)	
Caucasian, non-Hispanic	100%
Diagnosis of Type-2 Diabetes	100%
Had Teleophthalmology Screening	50%
Duration of diabetes	
<5 years	40%
5-19 years	30%
20+ years	30%
Highest Level of Education	
College graduate	10%
Some college/tech school	30%
High school graduate or GED	35%
Some high school	15%
Grade 8 or less	10%
Health literacy (Single Item Literacy Screener)	
High	85%
Moderate	10%
Low	5%
Primary Care Providers (n=9)	
Male	77.8%
Training Background	
MD/DO	44.4%
PA-C	33.3%
DNP	11.1%
RN	11.1%
Years in Practice	
>10 years	77.8%
5-10 years	0%
0-5 years	22.2%
Have Referred Patients for Teleophthalmology	87.5%

For peer review only - http://bmjopen.bmj.com/site/about/guidelines.xhtml

Patient Barriers and Facilitators

Major patient barriers to teleophthalmology use included being unfamiliar with teleophthalmology, misconceptions about diabetic eye screening, and logistical challenges (Table 2).

Table 2. Patient and Primary Care Provider Barriers and Facilitators

Patients	Barriers	- Unfamiliar with teleophthalmology*<br>- Misconceptions about diabetic eye screening*<br>- Logistical challenges* (e.g. time, transportation, out-of-pocket cost)<br>- Eye problems requiring in-person exam (e.g. glasses or glaucoma)<br>- Anxiety about receiving bad news regarding their eyes
	Facilitators	- Recommendation from primary care provider*<br>- Convenience of teleophthalmology* (e.g. same-day scheduling, location, quick)<br>- Belief that diabetic eye screening is important for preventing vision loss<br>- Knowing that pupil dilation is usually not necessary Teleophthalmology is considered a high-quality service due to University affiliation
Primary Care Providers	Barriers	- Difficulty identifying when patients are due for diabetic eye screening*<br>- Unfamiliar with teleophthalmology*<br>- Time constraints (e.g. many competing tasks during clinic visit)<br>- Concerns about conflicts with local eye doctors<br>- Concerns about patients' barriers (e.g. out-of-pocket cost)
	Facilitators	- Ease of referral process and results communication*<br>- Perceived benefits to patients (e.g. convenience, cost)<br>- Improved patient adherence with diabetic eye screening<br>- Benefits to the healthcare organization (e.g. increased reimbursement for improved quality metrics)

*Top barrier or facilitator identified at patient or primary care provider stakeholder meeting

Major patient facilitators included a recommendation from the patient's PCP and factors related to convenience. Most patients were unfamiliar with teleophthalmology, which prevented

For peer review only - http://bmjopen.bmj.com/site/about/guidelines.xhtml

them from taking the initiative to seek a teleophthalmology referral from their PCP for diabetic eye screening. In addition, several patients demonstrated a limited understanding of diabetic eye disease and the importance of screening.

> "I don't see an advantage to getting [my eyes] checked every year, unless you are having issues…" (Patient 5)

Many patients expressed the belief that having "good" vision and the absence of visual symptoms indicated that they do not have diabetic eye disease. The advantage of identifying and treating the disease at earlier, asymptomatic stages was not well understood. Patients were often unaware that ongoing screening was needed because the risk of retinopathy increases over time. Some patients believed that having one diabetic eye screening that was negative, even if it was several years ago, was sufficient and that additional screening was unnecessary.

Interestingly, neither a detailed understanding of diabetic eye disease nor the purpose of screening was necessary for patient adherence if the patient believed that yearly diabetic eye screening was important. This belief was often attributed to a strong recommendation from their PCP, which was the most common reason patients reported for using teleophthalmology.

> "My doctor thinks [teleophthalmology], you know, is a good test. And for me that's pretty much all I need." (Patient 16)

A PCP's recommendation was a major patient motivator because of the high level of trust patients placed in their PCP. Patients reported that they obtained most of their health information

For peer review only - http://bmjopen.bmj.com/site/about/guidelines.xhtml

directly from their PCP as well as through the clinic's diabetes education programs and informational handouts. Some patients also described maintaining independence, continuing to enjoy hobbies, and caring for other family members as important reasons for protecting their vision through screening. Their PCP's recommendation to pursue teleophthalmology screening reinforced these personal values.

Teleophthalmology was frequently endorsed as being "quick, easy, and painless" (Patient 5). When comparing teleophthalmology to traditional, in-person eye exams, many patients appreciated that teleophthalmology was conveniently-located in the same building as their primary care provider and accommodated walk-in scheduling with short wait times. In addition, they described the imaging as highly efficient, often taking "less than 5 minutes" (Patient 19). Teleophthalmology was often preferred by patients because it was more comfortable than traditional eye exams since pharmacologic pupil dilation was usually not needed. Pharmacologic pupil dilation was reported as a significant barrier to obtaining traditional eye exams.

> "Look at it from my viewpoint. I'm upstairs at the doctor's office and he says, 'Maybe you should go get [an eye photo] done…' You walk downstairs… sit down for 10 minutes… you get it done and you go home… I mean, it's so simple." (Patient 17)

> "The last thing I want to do is lose my eyesight [from diabetes] and before [teleophthalmology], I wasn't big on going to the eye doctor…" (Patient 16)

> "I have a terrible time when they dilate my eyes. The light, it hurts so bad." (Patient 5)

For peer review only - http://bmjopen.bmj.com/site/about/guidelines.xhtml

Although teleophthalmology addresses many barriers to diabetic eye screening, several important challenges remain for patients in this older, rural population with diabetes, many of whom live on a limited income. Teleophthalmology is only available in one primary care clinic so patients at other clinic locations were required to travel from their regular clinic to use it (median travel distance: 13.8 miles, range: 9.2-23.1 miles). Most patients also reported the need to pay out-of-pocket as a barrier due to limited insurance coverage for teleophthalmology.

PCP Barriers and Facilitators

Major PCP barriers to teleophthalmology use included difficulties identifying when patients are due for diabetic eye screening and being unfamiliar with teleophthalmology, while major facilitators were the ease of the referral process and results communication (Table 2). PCPs reported that they often did not have access to patients' eye records since all eye care providers in this community practice outside their health system and utilize different electronic health records (EHRs). Thus, PCPs depend on eye care providers to send diabetic eye screening documentation, which is not consistently performed. PCPs reported insufficient reminders, time, and resources to "track down" eye records as well as to discuss and refer patients for teleophthalmology as significant barriers. For example, enlisting the help of a medical assistant to request records from the patient's eye doctor was difficult due to time constraints during a typical PCP appointment. When a PCP cannot easily find these records, they would usually rely on the patient's self-reported date of their last diabetic eye screening, which has limited accuracy.

> "I don't have an easy way in my electronic records to see if [patients] had [diabetic eye screening] done and the patients never remember exactly when [it was done] ..." (PCP 7)

For peer review only - http://bmjopen.bmj.com/site/about/guidelines.xhtml

Faced with so many competing demands, PCPs often prioritized more urgent medical issues over teleophthalmology.

> "[As a provider] you are covering so many things… the foot exam, checking their cholesterol… [patients say] they saw the eye doctor and then you just go on to something else…" (PCP 4)

PCPs also reported being unfamiliar with teleophthalmology, which made some hesitant to refer patients. Some PCPs also worried about potential conflicts with local eye doctors and preferred to refer patients to eye doctors whom PCPs felt would be better suited to judge whether teleophthalmology would be appropriate.

> "I guess I just don't want to refer [a patient] in error if it's not really what the service is meant for." (PCP 3)

> "[I]… tend to refer patients to the ophthalmologists and they would probably be the ones to decide whether they were… teleophthalmology candidates" (PCP 5).

The most important facilitators for PCPs were the ease of referral process and results communication.

For peer review only - http://bmjopen.bmj.com/site/about/guidelines.xhtml

"[Teleophthalmology] was probably one of the easiest referrals I've done, and the turnaround [time for receiving results] was by far the best... I've had" (PCP 3).

Not only did PCPs describe teleophthalmology as being convenient for their patients, but also endorsed it for making it easier for PCPs to document referrals and reliably receive reports in their EHR among patients who had received diabetic eye screening.

A Model to Understand Patient and PCP Barriers in the Teleophthalmology Referral Process

Based on our results, we developed a model for understanding where key patient and PCP barriers occur during the primary care teleophthalmology referral process (Figure 1). PCPs are expected to initiate the referral process, but have difficulty remembering to ask patients about diabetic eye screening and identifying when patients are due. Next, they may have limited time to generate the referral and explain its purpose to the patient, especially when both the patient and the PCP are less familiar with teleophthalmology. Finally, referred patients may choose not to participate in teleophthalmology due to being unfamiliar with the technology, misconceptions or a lack of understanding about the importance of diabetic eye screening, and/or logistical difficulties related to time, travel or cost.

Possible Implementation Strategies

Patients and PCPs described possible implementation strategies to increase teleophthalmology use, which were categorized using the *Chronic Care Model* (Table 3). A variety of system-based implementation strategies were identified, primarily targeting the health system by streamlining PCP and clinic staff workflow processes (Table 3).

For peer review only - http://bmjopen.bmj.com/site/about/guidelines.xhtml

Table 3. Strategies to Increase Teleophthalmology Use Mapped to Chronic Care Model (CCM)

CCM Component	Target	Examples of Strategies
Health System Organization & Delivery System Design	Health System, PCPs, and Clinic Staff	- Workflow changes including clinic staff checklists and delegation of referrals†<br>- Provider/staff training to increase familiarity with teleophthalmology†<br>- Convenient scheduling and location†<br>- Provide financial incentives to individual PCPs for diabetic eye screening performance
Decision Support & Clinical Information Systems	Health System, PCPs, and Clinic Staff	- Best practice alert in EHR when patient is due for diabetic eye screening†<br>- Streamline processes for getting diabetic eye screening documentation into EHR†<br>- Provide PCPs with feedback/data on diabetic eye screening performance (e.g. quarterly)<br>- Generate lists of patients due for diabetic eye screening for clinic staff to contact
Self-Management Support & Community Resources	Patients, Families, Community Members, and Clinic Staff	- Patient education materials provided at primary care clinic visits†<br>- Increase education about diabetic eye screening in diabetes self-management classes†<br>- Publicize teleophthalmology services† (e.g. local media ad community health fairs)<br>- PCP clinic staff facilitate diabetic eye screening by calling patients and sending letters when due

†Strategies that can address top barriers or facilitators from Table 2

For peer review only

In contrast, fewer patient-directed strategies were identified. Strategies that addressed top barriers or facilitators included best practice alerts in the EHR to remind PCPs when patients are due for diabetic eye screening, improving PCP access to diabetic eye screening records, and clinic workflow changes such as the delegation of teleophthalmology referrals to clinic staff. In addition, arranging the scheduling and location for teleophthalmology imaging to maximize patient convenience was emphasized. Many PCPs recommended providing further training to

For peer review only - http://bmjopen.bmj.com/site/about/guidelines.xhtml

both providers and clinic staff on the use and purpose of teleophthalmology. Patients reported that greater education about the importance of diabetic eye screening, particularly to address common misconceptions, and increasing community awareness of teleophthalmology services would increase their likelihood of using teleophthalmology. Many patients preferred receiving written patient education materials from their PCP clinic or through presentations in diabetes education classes. They also recommended publicizing teleophthalmology through local newspaper or television advertisements.

CONCLUSIONS

Teleophthalmology is effective for increasing diabetic eye screening rates in rural populations.[13-17] However, there may be poor early adoption of this technology or a lack of sustained use by patients and PCPs despite having both equipment and trained personnel readily available—and even when the service is provided at no cost to the patient.[12] In this qualitative study, we identified patient and provider barriers, facilitators, and implementation strategies to improve and sustain teleophthalmology use over time for diabetic eye screening in a rural, multi-payer primary care setting.

Top patient barriers include being unfamiliar with teleophthalmology, misconceptions about diabetic eye screening, and logistical challenges, which were consistent with prior studies examining patient barriers to teleophthalmology[30-31] and traditional methods for diabetic eye screening.[32-39] Patients reported that the most common reason they obtained screening was a strong recommendation from their PCP. A PCP's recommendation for teleophthalmology was also found to be important in a few other qualitative studies, but was often not assessed in studies utilizing patient surveys.[37] Most studies found that patients were primarily motivated to seek

For peer review only - http://bmjopen.bmj.com/site/about/guidelines.xhtml

diabetic eye screening to prevent vision loss,[33-35, 37, 39] but this was less frequently reported by patients in our study. We found that many patients had limited knowledge and understanding about the purpose of diabetic eye screening, yet still obtained teleophthalmology screening when recommended by their PCP.

Our model demonstrates that the PCP initiates the teleophthalmology referral and that the patient's role is further downstream. Therefore, if the PCP is not reminded to consider making the referral, the patient may not obtain teleophthalmology regardless of how much education the patient has received. In addition, some PCPs reported concerns about the potential conflict between providing teleophthalmology in primary care and reduced referrals to local eye doctors, but studies have shown that teleophthalmology actually increases patient utilization of local eye care services by bringing into care many patients who would not otherwise obtain yearly eye exams.[40-41]. While patient education is important, our model suggests that implementation strategies to address PCP barriers may be more effective for increasing teleophthalmology use because of the PCP's primary role in the referral process. This is supported by studies that showed no significant differences in demographics or beliefs among patients who are or are not adherent with diabetic eye screening.[39] Instead, our data suggest that PCPs and clinic staff characteristics may be more influential in determining whether patients adhere with screening.

System-level implementation strategies are needed to provide PCPs with the information needed to make appropriate referrals, including better communication with eye care providers (e.g. up-to-date documentation of diabetic eye exams) and other EHR enhancements (e.g. systematic reminders when patients are due for screening and streamlining the referral process).[32,35] Addressing logistical barriers for patients by providing same-day, walk-in scheduling at a convenient location and minimizing out-of-pocket costs are also important. In

For peer review only - http://bmjopen.bmj.com/site/about/guidelines.xhtml

addition, PCP, clinic staff, and patient education as well as publicizing teleophthalmology throughout the community were also recommended by interview participants. Of note, we did not ask study participants to differentiate between strategies they believed would increase initial adoption or sustain use of teleophthalmology over time. Determining which of the strategies they identified would be useful for one or both purposes should be evaluated in future studies.

Limitations of our study include that all patients had some experience with diabetic eye screening. Patients who are not adherent with diabetes eye screening guidelines may have been less willing to participate. However, prior studies have shown that adherent and non-adherent patients experience similar barriers.[39] Our teleophthalmology program requires PCP referral and different barriers may occur in teleophthalmology programs allowing patient self-referral. Most patients in this study self-reported high levels of health literacy (85%), which was greater than that reported by rural adults from a similar population (70.9%).[30] In addition, all patients were Caucasian and native English speakers, which was representative of this rural community. A study of predominantly Latino and African-American patients in an urban, safety-net clinic found gaps in patient knowledge of diabetic retinopathy and screening, which prevented them from using teleophthalmology.[31] However, that study did not examine other factors that may limit urban patients' utilization of teleophthalmology. Further studies among patients from urban, other ethnic group, and non-native English-speaking backgrounds are needed to assess the generalizability of our findings to these populations.

Our study is one of the first to directly identify implementation strategies suggested by patients and PCPs that address their barriers and facilitators to using teleophthalmology for diabetic eye screening. In this rural, multi-payer health system, PCPs initiate teleophthalmology referrals and patients reported that a strong recommendation from their PCP was the most

For peer review only - http://bmjopen.bmj.com/site/about/guidelines.xhtml

important motivator for obtaining screening. Thus, system-level implementation strategies focusing on the PCP and clinic staff workflow processes appear to have the greatest potential to increase and maintain utilization in this setting. Further, larger studies are needed to evaluate the impact of these suggested strategies on increasing teleophthalmology use and diabetic eye screening rates.

Teleophthalmology can substantially increase diabetic eye screening rates and prevent blindness. While this technology addresses numerous logistical barriers to diabetic eye screening, we found several additional barriers to its use by patients and PCPs. System-based implementation strategies primarily targeting PCP barriers in conjunction with improved patient and provider education may increase teleophthalmology use in rural, multi-payer primary care clinics.

For peer review only

ACKNOWLEDGEMENTS

The authors acknowledge the UW ICTR-CAP Qualitative Research Group (QRG), Wisconsin Network for Research Support (WINRS), Community Advisors on Research Design and Strategies (CARDS)®, and the Primary Care Academics Transforming Healthcare (PATH) Writing Collaborative for their feedback on interview guides and recruitment methodology. We thank the Mile Bluff Medical Center Diabetes Patient Advisory Council and Diabetes Quality Care Team for providing feedback to refine our research results.

Prior abstract publication: This study was presented at the 2017 Association for Research in Vision and Ophthalmology (ARVO) Annual Meeting in Baltimore, MD, USA.

Author contributions: Y.L., R.S., and N.J. developed the interview guides. R.S. performed the interviews. Y.L., N.Z., J.C., and N.J. analyzed the data. J.M., R.K., T.B., and M.S. contributed to the discussion and reviewed/edited the manuscript. Y.L. and N.Z. wrote the manuscript.

Guarantor statement: Dr. Liu accepts full responsibility for this work as a whole, including the study design, access to the data, and the decision to submit and publish the manuscript.

For peer review only

For peer review only - http://bmjopen.bmj.com/site/about/guidelines.xhtml

Figure Legends (in order of appearance in the text)

Table 1. Patient and Primary Care Provider Demographics

Table 2. Patient and Primary Care Provider Barriers and Facilitators

Figure 1. Barriers in the Teleophthalmology Referral Process

Table 3. Strategies to Increase Teleophthalmology Use Mapped to Chronic Care Model (CCM)

For peer review only

For peer review only - http://bmjopen.bmj.com/site/about/guidelines.xhtml

REFERENCES

1. National diabetes statistics report: estimates of diabetes and its burden in the United States, 2014. *Atlanta, GA: US Department of Health and Human Services* 2014;2014

2. National Eye Institute: eye disease statistics fact sheet. *National Institutes of Health*, 2015. Available from https://nei.nih.gov/sites/default/files/nei-pdfs/NEI_Factsheet_July_2015_v2.pdf

3. Group ETDRSR. Early photocoagulation for diabetic retinopathy: ETDRS report number 9. *Ophthalmology* 1991;98(5):766-85.

4. National diabetes fact sheet: national estimates and general information on diabetes and prediabetes in the United States, 2010. *http://www.caldiabetes.org/content_display.cfm?FontSize= normal&contentID= 562&CategoriesID= 31*

5. Lee PP, Feldman ZW, Ostermann J, et al. Longitudinal rates of annual eye examinations of persons with diabetes and chronic eye diseases. *Ophthalmology* 2003;110(10):1952-59.

6. Scanlon PH. The English National Screening Programme for diabetic retinopathy 2003-2016. *Acta Diabetologica* 2017;54(6):515-25. doi: 10.1007/s00592-017-0974-1

7. Davidson JA. The Increasing Role of Primary Care Physicians in Caring for Patients With Type 2 Diabetes Mellitus. *Mayo Clinic Proceedings* 2010;85(12 Suppl):S3-S4. doi: 10.4065/mcp.2010.0466

8. Boyle JP, Thompson TJ, Gregg EW, et al. Projection of the year 2050 burden of diabetes in the US adult population: dynamic modeling of incidence, mortality, and prediabetes prevalence. *Population health metrics* 2010;8(1):29.

9. Liew G, Michaelides M, Bunce C. A comparison of the causes of blindness certifications in England and Wales in working age adults (16–64 years), 1999–2000 with 2009–2010. *BMJ open* 2014;4(2):e004015.

10. Whited JD, Datta SK, Aiello LM, et al. A modeled economic analysis of a digital teleophthalmology system as used by three federal healthcare agencies for detecting proliferative diabetic retinopathy. *Telemedicine Journal & E-Health* 2005;11(6):641-51.

11. Garg S, Zimmer-Galler I. Retinal Physician - Telemedicine and Remote Imaging for Diabetic Retinopathy Evaluation: An Update. *Retinal Physician* 2013;10(March 2013):49-51.

12. Mansberger SL, Sheppler C, Barker G, et al. Long-term comparative effectiveness of telemedicine in providing diabetic retinopathy screening examinations: a randomized clinical trial. *JAMA ophthalmology* 2015;133(5):518-25.

For peer review only - http://bmjopen.bmj.com/site/about/guidelines.xhtml

13. Krishna S, Gillespie KN, McBride TM. Diabetes burden and access to preventive care in the rural United States. *The Journal of Rural Health* 2010;26(1):3-11.

14. Dansky KH, Dirani R. The use of health care services by people with diabetes in rural areas. *The Journal of Rural Health* 1998;14(2):129-37.

15. Leese GP, Ahmed S, Newton RW, et al. Use of mobile screening unit for diabetic retinopathy in rural and urban areas. *BMJ* 1993;306(6871):187-89.

16. Cummings DM, Morrissey S, Barondes MJ, et al. Screening for diabetic retinopathy in rural areas: the potential of telemedicine. *The Journal of Rural Health* 2001;17(1):25-31.

17. Ahmed MD, Petrany SM, Fry II, et al. Screening diabetic and hypertensive patients for ocular pathology using telemedicine technology in rural West Virginia: a retrospective chart review. *West Virginia Medical Journal* 2013;109(1):6-10.

18. Hussey P, Anderson GF. A comparison of single-and multi-payer health insurance systems and options for reform. *Health policy* 2003;66(3):215-28.

19. Chedid EH, Golden QR, Jager RD. Operational challenges in delivery of a charity care program for diabetic retinopathy screening in an urban setting. *The Permanente Journal* 2013;17(1):21.

20. Arora S, Kurji AK, Tennant MTS. Dismantling sociocultural barriers to eye care with tele-ophthalmology: lessons from an Alberta Cree community. *Clinical & Investigative Medicine* 2013;36(2):57-63.

21. Sofaer S. Qualitative methods: what are they and why use them? *Health services research* 1999;34(5 Pt 2):1101.

22. Li HK, Horton M, Bursell S-E, et al. Telehealth practice recommendations for diabetic retinopathy. *Telemedicine and e-health* 2011;17(10):814-37.

23. Bodenheimer T, Wagner EH, Grumbach K. Improving primary care for patients with chronic illness. *JAMA* 2002;288(14):1775-79.

24. Bodenheimer T, Wagner EH, Grumbach K. Improving Primary Care for Patients With Chronic Illness: The Chronic Care Model, Part 2. *JAMA* 2002;288(15):1909-14.

25. Sandelowski M. Sample size in qualitative research. *Research in Nursing & Health* 1995;18(2):179-83. doi: 10.1002/nur.4770180211

26. Birt L, Scott S, Cavers D, et al. Member checking: A tool to enhance trustworthiness or merely a nod to validation? *Qualitative Health Research* 2016;26(13):1802-11.

For peer review only

For peer review only - http://bmjopen.bmj.com/site/about/guidelines.xhtml

27. Delbecq AL, Van de Ven AH, Gustafson DH. Group techniques for program planning: A guide to nominal group and Delphi processes: Scott Foresman 1975.

28. O'Brien BC, Harris IB, Beckman TJ, et al. Standards for Reporting Qualitative Research: A Synthesis of Recommendations. *Academic Medicine* 2014;89(9):1245-51. doi: 10.1097/ACM.0000000000000388

29. Morris NS, MacLean CD, Chew LD, et al. The Single Item Literacy Screener: evaluation of a brief instrument to identify limited reading ability. *BMC family practice* 2006;7(1):21.

30. Zahnd WE, Scaife SL, Francis ML. Health Literacy Skills in Rural and Urban Populations. *American Journal of Health Behavior* 2009;33(5):550-57. doi: 10.5993/AJHB.33.5.8

31. George SM, Hayes EM, Fish A, et al. Understanding the Knowledge Gap Experienced by U.S. Safety Net Patients in Teleretinal Screening. *AMIA Annual Symposium Proceedings* 2017;2016:590-99.

32. Hartnett ME, Key IJ, Loyacano NM, et al. Perceived Barriers to Diabetic Eye Care: Qualitative Study of Patients and Physicians. *Archives of Ophthalmology* 2005;123(3):387-91. doi: 10.1001/archopht.123.3.387

33. Glasson NM, Larkins SL, Crossland LJ. What do patients with diabetes and providers think of an innovative Australian model of remote diabetic retinopathy screening? A qualitative study. *BMC Health Services Research* 2017;17:158.

34. Lewis K, Patel D, Yorston D, et al. A Qualitative Study in the United Kingdom of Factors Influencing Attendance by Patients with Diabetes at Ophthalmic Outpatient Clinics. *Ophthalmic Epidemiology* 2007;14(6):375-80.

35. Ellish NJ, Royak-Schaler R, Passmore SR, et al. Knowledge, Attitudes, and Beliefs about Dilated Eye Examinations among African-Americans. *Investigative Ophthalmology & Visual Science* 2007;48(5):1989-94. doi: 10.1167/iovs.06-0934

36. Holley CD, Lee PP. Primary Care Provider Views of the Current Referral-to-Eye-Care Process: Focus Group Results. *Investigative Ophthalmology & Visual Science* 2010;51(4):1866-72. doi: 10.1167/iovs.09-4512

37. van Eijk KND, Blom JW, Gussekloo J, et al. Diabetic retinopathy screening in patients with diabetes mellitus in primary care: Incentives and barriers to screening attendance. *Diabetes Research and Clinical Practice* 2012;96(1):10-16.

38. Lindenmeyer A, Sturt JA, Hipwell A, et al. Influence of primary care practices on patients' uptake of diabetic retinopathy screening: a qualitative case study. *Br J Gen Pract* 2014;64(625):e484-e92. doi: 10.3399/bjgp14X680965

For peer review only - http://bmjopen.bmj.com/site/about/guidelines.xhtml

39. Hipwell AE, Sturt J, Lindenmeyer A, et al. Attitudes, access and anguish: a qualitative interview study of staff and patients' experiences of diabetic retinopathy screening. *BMJ open* 2014;4(12):e005498.

40. Owsley C, McGwin G, Lee DJ, et al. Diabetes eye screening in urban settings serving minority populations: detection of diabetic retinopathy and other ocular findings using telemedicine. *JAMA ophthalmology* 2015;133(2):174-81.

41. Kirkizlar E, Serban N, Sisson JA, et al. Evaluation of Telemedicine for Screening of Diabetic Retinopathy in the Veterans Health Administration. *Ophthalmology* 2013;120(12):2604-10. doi: 10.1016/j.ophtha.2013.06.029

For peer review only

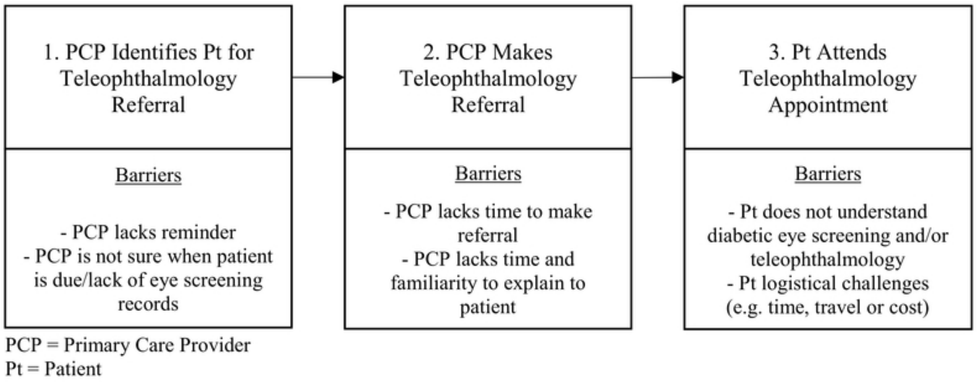

Figure 1. Barriers in the Teleophthalmology Referral Process

68x27mm (300 x 300 DPI)

Supplemental Appendix S1. Patient Interview Guide

Introduction:

Thank you so much for taking the time to meet with me. Our research team at the University of Wisconsin is working with the Mile Bluff Medical Center to learn more about patient experiences with diabetic eye exams.

You were invited to participate because you are a Mile Bluff Medical Center patient who has diabetes. I expect our conversation will last about 45 minutes. At the end of our discussion, I have a few questions about your background. Participation in this interview is voluntary. You can stop the interview at any time and if there are any questions you don't want to answer you can just tell me to skip those. Everything you tell me today will be kept confidential. Only our research team led by Dr. Yao Liu, a UW eye doctor, will have access to this information.

I will be audio recording this interview so that I can review our discussion later and make sure I accurately get all the ideas and opinions that you share. This interview is about <u>your</u> personal experience with eye exams. There are no "right" or "wrong" answers.

Do you have any questions before we begin? If you think of any questions as we go along, feel free to ask them any time.

Part I. Interview Questions

First, I'd like to ask you some general questions about how you take care of your diabetes.

What are things you do each day that are related to your diabetes?

Prompts:

- ☐ There are many things people do to take care of their diabetes. What do you see as the biggest priorities for you?
- ☐ *[if none]* What are some of the things your doctor may have discussed with you that may be important for people with diabetes to do?
- ☐ Are there things that you watch out for that tell you your diabetes may be getting worse?

Now I'd like to show you some pictures. These photos show two ways of doing eye checks for people with diabetes. The top photo shows a Traditional Eye Exam, where an eye doctor uses eye drops to dilate and examine your eyes. The bottom photo shows an Eye Photo Test, where a technician uses a special camera to take photos of your eyes.

Some people get yearly eye checks as part of their diabetes care. What do you think are the advantages of this? What are possible disadvantages to getting these eye checks?

Prompts:

- ☐ How do you know when it is time for you to get an eye check for diabetes?
- ☐ Why did you choose to get your eyes checked?

In your experience, how easy is it to regularly get diabetic eye checks as compared to *[use their example about something they do for their diabetes]*?

Prompts:

- ☐ Can you tell me more about why you find it easier/harder?

For peer review only - http://bmjopen.bmj.com/site/about/guidelines.xhtml

- ☐ You mentioned *[x]* as one thing that makes it hard to regularly get eye exams. Can you tell me more about that? What else might make it hard?
- ☐ What do you think might make it easier for you or other people with diabetes in your community to regularly get eye exams?

How do you find information about diabetic eye checks?

Prompts:

- ☐ From where do you get this information?
- ☐ Are there helpful resources here in your community?
- ☐ What support or resources do you think would help people learn more about this or help them get their eyes checked?
- ☐ *[If they don't currently get information about eye exams.]* Where do you get information about what you need to do related to diabetes?

[If patient has not had teleophthalmology, jump to page 4: Version II. Clinical Eye Exam]

Version I. Teleophthalmology

I believe you had the Eye Photo Test *[point to photo]* ***[x]*** **weeks/months ago, is this correct?**

Tell me about your experience starting with how you first learned about the Eye Photo Test.

Prompts:

- ☐ Do you remember who explained this test to you?
- ☐ What did they tell you about it?
- ☐ Did you get all the information you wanted?
- ☐ What made you decide to have the Eye Photo Test?
- ☐ Why do you think your doctor referred you for the Eye Photo Test?

Next, I have a few questions about scheduling the Eye Photo Test and getting to your appointment.

How easy was it to schedule the Eye Photo Test?

Prompts:

- ☐ How long did you wait between the time you scheduled the Eye Photo Test and when you had the appointment? (e.g. a few days, weeks or months)
- ☐ How easy was it to get to the clinic for the photo eye test? Did you drive yourself or did someone else drive you? How long did it take to get from your home to the clinic?

Now I'd like you to tell me about your experience with the Eye Photo Test itself.

Prompts:

- ☐ About how long did the Eye Photo Test take?
- ☐ Did you receive results from your Eye Photo Test?
- ☐ *[If YES]* How quickly did you get those results? Is there anything that could be changed or improved about the way you receive the results?
- ☐ *[If NO]* Did you want to receive the results from the Eye Photo Test?
- ☐ How would you like to receive your Eye Photo Test results? (e.g. letter in the mail or phone call)

How would you explain the Eye Photo Test to a friend or family member?

Prompts:

- ☐ Would you be willing to take the Eye Photo Test again? Why or why not?

For peer review only

Do you think that there is anything that could be improved about your experience with the Eye Photo Test?

Prompts:

- ☐ How can we make the Eye Photo Test more available to other people in your community who have diabetes?
- ☐ For your community, do you think $20 is a reasonable cost for the Eye Photo Test?

Do you plan to have another diabetic eye check in the future?

Prompts:

- ☐ *[If YES]* How soon and where might you schedule this? [verify what type of exam – Traditional Eye Exam vs Eye Photo Test]
- ☐ *[If NO]* Why not?

Let's look at the picture of the Traditional Eye Exam *[point to photo]***. Have you ever had this exam where an eye doctor uses eye drops to dilate and examine your eyes?**

Prompts:

- ☐ *[If YES]* Tell me about your experience with this exam. What was it like?

Does your insurance cover Traditional Eye Exams? How much do you normally pay to have a Traditional Eye Exam?

Let's look at the picture of the two types of eye checks again. If you had to choose between having your eyes checked using a Traditional Eye Exam or an Eye Photo Test, which would you prefer? Why?

[Jump to Part II on page 5]

For peer review only

Version II. Clinical Eye Exam

I believe you have not had the Eye Photo Test *[point to photo]*, **is this correct?**

Now, let's look at the picture of the Traditional Eye Exam *[point to photo]*. **Have you ever had this exam where an eye doctor uses eye drops to dilate and examine your eyes? If so, do you recall roughly the last time you had a Traditional Eye Exam?**

Next, I have a few questions about scheduling the Traditional Eye Exam and getting to your appointment.

How easy was it to schedule the Traditional Eye Exam?

Prompts:

- ☐ How long did you wait between the time you scheduled the exam and when you had the appointment? (e.g. a few days, weeks or months)
- ☐ How easy was it to get to the clinic for the Traditional Eye Exam? Did you drive yourself or did someone else drive you? How long did it take to get from your home to the clinic?

Now I'd like you to tell me about your experience with the Traditional Eye Exam itself.

Prompts:

- ☐ About how long did the exam take?
- ☐ Did you receive results from your Traditional Eye Exam?
- ☐ *[If YES]* How quickly did you get those results? Is there anything that could be changed or improved about the way you receive the results?
- ☐ *[If NO]* How would you want to receive the results from the exam?

How would you describe the Traditional Eye Exam to a friend or family member?

Prompts:

- ☐ Would you be willing to have a Traditional Eye Exam again? Why or why not?

Do you plan to have another diabetic eye check in the future?

Prompts:

- ☐ *[If YES]* How soon and where might you schedule this? [verify what type of exam – Traditional Eye Exam vs Eye Photo Test]
- ☐ *[If NO]* Why not?

Does your insurance cover Traditional Eye Exams? How much do you normally pay to have a Traditional Eye Exam?

Let's look at the picture of the two types of eye exams again. If you had to choose between having your eyes examined using a Traditional Eye Exam or an Eye Photo Test, which would you prefer? Why?

For peer review only

Part II. Wrap-up and Demographics

Is there anything else you think is important for me to know about your experience with getting diabetic eye checks?

Thanks so much for all this great information. I really appreciate your sharing your experience with me.

As I mentioned earlier, there is just one last thing—a short list of questions to get some background information. Please understand that we only want this this information so that we have an accurate picture of who is involved in our study; we know the community is diverse and we want to be sure we hear many different perspectives and hear from people with a wide range of backgrounds. We are not making any judgments or assumptions about you based on this information.

I will read the questions to you or if you prefer, you can read the questions on your own.

[Read or hand patient page 6 to complete]

For peer review only

Patient Background Information

1. How long have you had diabetes?

a) Less than 5 years
b) Between 5-10 years
c) Between 10-15 years
d) More than 15 years

2. Do you drive yourself to your eye appointments? (If no, who drives you)?

a) Yes
b) No, my ______________________________ drives me to appointments

3. During regular clinic hours, are you easily able to get your eye appointments?

a) Yes
b) No

4. What level of school have you completed?

____

5. We know that health information is often written in a way that is complicated and hard to understand. How often do you need to have someone help you when you read instructions, pamphlets, or other written material from your doctor or pharmacy?

a) Never
b) Rarely
c) Sometimes
d) Often
e) Always

For peer review only

Supplemental Appendix S2. Primary Care Provider (PCP) Interview Guide

Introduction:

Thank you so much for taking the time to talk with me. I'm going to start off by giving you an introduction and our goals for this interview.

As you know, in collaboration with Mile Bluff Medical Center (MBMC), our research team at the University of Wisconsin is studying primary care provider perspectives on diabetic eye exams.

You are being invited to participate in this interview because we are interested in learning more about your experience, as a primary care provider, with teleophthalmology and your ideas on ways to make it easier for you to use. We are also conducting separate interviews with patients to understand their experiences as well.

Participation in this study is voluntary. You may discontinue your participation at any time. There won't be any identifying information linked with your responses. Only researchers on this project will have access to the data gathered.

I will audiotape this interview so that I can take fewer notes as we talk and review our conversation later to ensure that I don't miss any information. The interview should take about 15-20 minutes. Please feel free to stop me at any time with questions or concerns.

Do you have any questions about the study before we begin?

Version I. Teleophthalmology

[NOTE: If PCP has not referred patients for teleophthalmology, skip to page 3: Version II. Clinical eye exam]

Tell me about your experience with teleophthalmology.

Prompts:

- ☐ Why did you choose to refer patients for teleophthalmology?
- ☐ What helped in that process? What didn't help in that process?
- ☐ Can you describe an instance when a referral did not happen and why?

What makes referring patients for diabetic eye exams easier or more difficult than other health maintenance screenings (e.g. mammography and colonoscopy)?

Now I'd like to ask you to walk me through your decision to refer a typical patient for teleophthalmology.

Prompts:

- ☐ Do you refer the patient? Do you have help from medical assistants or schedulers?
- ☐ How do you decide whether to refer a patient for teleophthalmology versus a traditional diabetic eye exam?
- ☐ What patient factors, such as their characteristics or preferences, influence your decision?
- ☐ What about non-patient factors that might influence your decision, such as how busy you are in clinic?
- ☐ When you see a patient with multiple health issues, at what point during a typical clinic visit might you bring up the topic of diabetic eye screening?

For peer review only

How do you explain teleophthalmology to the patient?

Prompts:

- ☐ How do patients typically respond?
- ☐ What makes you feel confident, or not, that the patient will obtain teleophthalmology screening?
- ☐ Of your patients that have had this test done, what kind of feedback do they give you?

Is there anything that might make easier for you to have your patients use teleophthalmology?

Prompts:

- ☐ Some examples may include:
  Interventions to identify patients eligible for eye screening
  Delegation of referrals to support staff
  Walk-in/flexible scheduling of eye screening
- ☐ Do you have any concerns or questions about this technology?
- ☐ Are there incentives or processes in place that encourage referring patients for this or other types of screening?

This has been very helpful. Before we conclude our conversation, is there anything else I should be asking you about teleophthalmology or diabetic eye exams that is important for me to know?

Thank you very much for your participation!

For peer review only

Version II. Clinical eye exam *[if PCP has not referred patients for teleophthalmology]*

Tell me about your experience with teleophthalmology.

Prompts:

- ☐ Do you have any concerns or questions about this technology?
- ☐ Have you considered having patients get teleophthalmology screening instead of a clinical diabetic eye exam? Why or why not?

Is there anything that might make it more likely for you to have your patients use teleophthalmology?

Prompts:

- ☐ For example:
  Interventions to identify patients eligible for eye screening
  Delegation of referrals to support staff
  Walk-in/flexible scheduling of eye screening
- ☐ Are there incentives or processes in place that encourage you or your colleagues to refer patients for this or other types of screening?

What makes referring patients for diabetic eye exams easier or more difficult than other health maintenance screenings (e.g. mammography and colonoscopy)?

Now I'd like you to walk me through the steps you or your staff take when helping patients get diabetic eye exams.

Prompts:

- ☐ Do you refer the patient? Do you have help from medical assistants or schedulers? Do patients refer themselves?
- ☐ How do you decide whether to refer a patient for a diabetic eye exam?
- ☐ What patient factors, such as their characteristics or preferences, influence your decision?
- ☐ What about non-patient factors that might influence your decision, such as how busy you are in clinic?
- ☐ When you see a patient with multiple health issues, at what point during a typical clinic visit do you bring up the topic of diabetic eye screening?
- ☐ Is there anything that could be improved about this process?

How do you explain diabetic eye exams to patients?

Prompts:

- ☐ How do patients typically respond?
- ☐ What makes you feel confident, or not, that the patient will obtain the exam?
- ☐ Of your patients that have had a diabetic eye exam, what kind of feedback do they give you?

This has been very helpful. Before we conclude our conversation, is there anything else I should be asking you about teleophthalmology or diabetic eye exams that is important for me to know?

Thank you very much for your participation!

For peer review only

Reporting checklist for qualitative study.

Based on the SRQR guidelines.

Instructions to authors

Complete this checklist by entering the page numbers from your manuscript where readers will find each of the items listed below.

Your article may not currently address all the items on the checklist. Please modify your text to include the missing information. If you are certain that an item does not apply, please write "n/a" and provide a short explanation.

Upload your completed checklist as an extra file when you submit to a journal.

In your methods section, say that you used the SRQR reporting guidelines, and cite them as:

O'Brien BC, Harris IB, Beckman TJ, Reed DA, Cook DA. Standards for reporting qualitative research: a synthesis of recommendations. Acad Med. 2014;89(9):1245-1251.

		Reporting Item	Page Number
	#1	Concise description of the nature and topic of the study identifying the study as qualitative or indicating the approach (e.g. ethnography, grounded theory) or data collection methods (e.g. interview, focus group) is recommended	1
	#2	Summary of the key elements of the study using the abstract format of the intended publication; typically includes background, purpose, methods, results and conclusions	3-4
Problem formulation	#3	Description and signifcance of the problem / phenomenon studied: review of relevant theory and empirical work; problem statement	7-8
Purpose or research question	#4	Purpose of the study and specific objectives or questions	8
Qualitative approach and research paradigm	#5	Qualitative approach (e.g. ethnography, grounded theory, case study, phenomenolgy, narrative research)	9-12

For peer review only

		and guiding theory if appropriate; identifying the research paradigm (e.g. postpositivist, constructivist / interpretivist) is also recommended; rationale. The rationale should briefly discuss the justification for choosing that theory, approach, method or technique rather than other options available; the assumptions and limitations implicit in those choices and how those choices influence study conclusions and transferability. As appropriate the rationale for several items might be discussed together.	
Researcher characteristics and reflexivity	#6	Researchers' characteristics that may influence the research, including personal attributes, qualifications / experience, relationship with participants, assumptions and / or presuppositions; potential or actual interaction between researchers' characteristics and the research questions, approach, methods, results and / or transferability	11-12
Context	#7	Setting / site and salient contextual factors; rationale	8-9
Sampling strategy	#8	How and why research participants, documents, or events were selected; criteria for deciding when no further sampling was necessary (e.g. sampling saturation); rationale	10-11
Ethical issues pertaining to human subjects	#9	Documentation of approval by an appropriate ethics review board and participant consent, or explanation for lack thereof; other confidentiality and data security issues	12
Data collection methods	#10	Types of data collected; details of data collection procedures including (as appropriate) start and stop dates of data collection and analysis, iterative process, triangulation of sources / methods, and modification of procedures in response to evolving study findings; rationale	9-10
Data collection instruments and technologies	#11	Description of instruments (e.g. interview guides, questionnaires) and devices (e.g. audio recorders) used for data collection; if / how the instruments(s) changed over the course of the study	9-10

For peer review only

Units of study	#12	Number and relevant characteristics of participants, documents, or events included in the study; level of participation (could be reported in results)	12-13
Data processing	#13	Methods for processing data prior to and during analysis, including transcription, data entry, data management and security, verification of data integrity, data coding, and anonymisation / deidentification of excerpts	11-12
Data analysis	#14	Process by which inferences, themes, etc. were identified and developed, including the researchers involved in data analysis; usually references a specific paradigm or approach; rationale	11-12
Techniques to enhance trustworthiness	#15	Techniques to enhance trustworthiness and credibility of data analysis (e.g. member checking, audit trail, triangulation); rationale	11-12
Syntheses and interpretation	#16	Main findings (e.g. interpretations, inferences, and themes); might include development of a theory or model, or integration with prior research or theory	14-21
Links to empirical data	#17	Evidence (e.g. quotes, field notes, text excerpts, photographs) to substantiate analytic findings	14-21
Integration with prior work, implications, transferability and contribution(s) to the field	#18	Short summary of main findings; explanation of how findings and conclusions connect to, support, elaborate on, or challenge conclusions of earlier scholarship; discussion of scope of application / generalizability; identification of unique contributions(s) to scholarship in a discipline or field	21-24
Limitations	#19	Trustworthiness and limitations of findings	23
Conflicts of interest	#20	Potential sources of influence of perceived influence on study conduct and conclusions; how these were managed	6
Funding	#21	Sources of funding and other support; role of funders in data collection, interpretation and reporting	5-6

The SRQR checklist is distributed with permission of Wolters Kluwer © 2014 by the Association of American Medical Colleges. This checklist can be completed online using

For peer review only

https://www.goodreports.org/, a tool made by the EQUATOR Network in collaboration with Penelope.ai

For peer review only

BMJ Open

Identification of Barriers, Facilitators, and System-based Implementation Strategies to Increase Teleophthalmology Use for Diabetic Eye Screening in a Rural U.S. Primary Care Clinic: A Qualitative Study

Journal:	*BMJ Open*
Manuscript ID	bmjopen-2018-022594.R1
Article Type:	Research
Date Submitted by the Author:	09-Oct-2018
Complete List of Authors:	Liu, Yao; University of Wisconsin-Madison School of Medicine and Public Health, Ophthalmology and Visual Sciences; University of Wisconsin-Madison School of Medicine and Public Health, Health Innovation Program<br>Zupan, Nicholas; University of Wisconsin-Madison School of Medicine and Public Health, Ophthalmology and Visual Sciences<br>Swearingen, Rebecca; University of Wisconsin-Madison School of Medicine and Public Health, Ophthalmology and Visual Sciences<br>Jacobson , Nora; University of Wisconsin Madison Institute for Clinical and Translational Research<br>Carlson, Julia; University of Wisconsin-Madison School of Medicine and Public Health, Ophthalmology and Visual Sciences<br>Mahoney, Jane E. ; University of Wisconsin School of Medicine and Public Health, Medicine<br>Klein, Ronald; University of Wisconsin-Madison School of Medicine and Public Health, Ophthalmology and Visual Sciences<br>Bjelland, Timothy; Mile Bluff Medical Center<br>Smith, Maureen; University of Wisconsin School of Medicine and Public Health, Health Innovation Program; University of Wisconsin School of Medicine and Public Health, Population Health Sciences, Family Medicine and Community Health
**Primary Subject Heading**:	Ophthalmology
Secondary Subject Heading:	Health services research, Qualitative research, General practice / Family practice
Keywords:	Diabetic retinopathy < DIABETES & ENDOCRINOLOGY, Organisation of health services < HEALTH SERVICES ADMINISTRATION & MANAGEMENT, PUBLIC HEALTH, Telemedicine < BIOTECHNOLOGY & BIOINFORMATICS, QUALITATIVE RESEARCH

Title: Identification of Barriers, Facilitators, and System-based Implementation Strategies to Increase Teleophthalmology Use for Diabetic Eye Screening in a Rural U.S. Primary Care Clinic: A Qualitative Study

Running Title: Barriers, Facilitators, and System-based Strategies for Teleophthalmology

Authors: Liu, Yao M.D.[1,2]; Zupan, Nicholas J. M.P.H.[1]; Swearingen, Rebecca B.S.[1,2]; Jacobson, Nora A. Ph.D.[3]; Carlson, Julia A. B.S.[1]; Mahoney, Jane E. M.D.[4]; Klein, Ronald M.D. M.P.H.[1]; Bjelland, Timothy D. D.O.;[5] Smith, Maureen A. M.D. M.P.H. Ph.D.[2,6]

Affiliations:

[1]University of Wisconsin School of Medicine and Public Health, Department of Ophthalmology and Visual Sciences

[2]University of Wisconsin School of Medicine and Public Health, Health Innovation Program

[3]University of Wisconsin School of Medicine and Public Health, Institute for Clinical and Translational Research

[4]University of Wisconsin School of Medicine and Public Health, Department of Medicine

[5]Mile Bluff Medical Center

[6]University of Wisconsin School of Medicine and Public Health, Departments of Population Health Sciences, Family Medicine and Community Health

Corresponding Author:

For peer review only - http://bmjopen.bmj.com/site/about/guidelines.xhtml

For peer review only

Yao Liu, MD

Assistant Professor, Department of Ophthalmology and Visual Sciences

University of Wisconsin School of Medicine and Public Health

2870 University Ave, Ste. 206

Madison, WI 53705

Phone: 608-263-1481

Fax: 608-263-7694

Email: liu463@wisc.edu

Word Count: 4,921

Total Number of Tables and Figures: 4

Keywords (up to 5): implementation, diabetic eye screening, multi-payer, telemedicine, qualitative

For peer review only

ABSTRACT

Objective

Teleophthalmology for diabetic eye screening is an evidence-based intervention substantially underutilized in U.S multi-payer primary care clinics, even when equipment and trained personnel are readily available. We sought to identify patient and primary care provider (PCP) barriers, facilitators, as well as strategies to increase teleophthalmology use.

Design

We conducted standardized open-ended, individual interviews and analyzed the transcripts using both inductive and directed content analysis to identify barriers and facilitators to teleophthalmology use. The *Chronic Care Model* was used as a framework for development of the interview guide and for categorizing implementation strategies to increase teleophthalmology use.

Setting

A rural, U.S. multi-payer primary care clinic with an established teleophthalmology program for diabetic eye screening.

Participants

We conducted interviews with 29 participants (20 patients with diabetes and 9 PCPs).

Results

For peer review only - http://bmjopen.bmj.com/site/about/guidelines.xhtml

Major patient barriers to teleophthalmology use included being unfamiliar with teleophthalmology, misconceptions about diabetic eye screening, and logistical challenges. Major patient facilitators included a recommendation from the patient's PCP and factors related to convenience. Major PCP barriers to referring patients for teleophthalmology included difficulty identifying when patients are due for diabetic eye screening and being unfamiliar with teleophthalmology. Major PCP facilitators included the ease of the referral process and communication of screening results. Based on our results, we developed a model that maps where these key patient and PCP barriers occur in the teleophthalmology referral process. Patients and PCPs also identified implementation strategies to directly address barriers and facilitators to teleophthalmology use.

Conclusions

Patients and PCPs have limited familiarity with teleophthalmology for diabetic eye screening. PCPs were expected to initiate teleophthalmology referrals, but reported significant difficulty identifying when patients are due for diabetic eye screening. System-based implementation strategies primarily targeting PCP barriers in conjunction with improved patient and provider education may increase teleophthalmology use in rural, U.S. multi-payer primary care clinics.

For peer review only - http://bmjopen.bmj.com/site/about/guidelines.xhtml

Article Summary

Strengths and limitations of this study:

- We used qualitative methods to capture the real-world perspectives of patients and providers regarding barriers and facilitators to teleophthalmology use in a rural U.S. multi-payer primary care clinic with an active teleophthalmology program.
- We identified and categorized implementation strategies directly suggested by patients and providers using the *Chronic Care Model*.
- All patients were Caucasian and native English speakers.
- Most patients in this study self-reported high levels of general health literacy (85%), which was greater than that reported by rural adults from a similar population (70.9%).
- We did not systematically assess patient knowledge of diabetic eye screening.

Funding statement

This work was supported by NIH/NEI K23 EY026518-02 and a Wisconsin Partnership Program New Investigator Award. It was also supported, in part, by an institutional grant from Research to Prevent Blindness, Inc., New York, NY, USA to the University of Wisconsin School of Medicine and Public Health, Dept. of Ophthalmology and Visual Sciences. Additional support came from the Clinical and Translational Science Award (CTSA) program, through the NIH National Center for Advancing Translational Sciences (NCATS), grant UL1TR000427. Further support was received from the University of Wisconsin (UW) School of Medicine and Public Health Innovation Program, the Wisconsin Partnership Program, and the UW Institute for Clinical and Translational Research-Community Academic Partnerships (ICTR-CAP) core.

For peer review only - http://bmjopen.bmj.com/site/about/guidelines.xhtml

The study sponsors had no role in the design and conduct of the study; collection, management, analysis, and interpretation of the data; preparation, review, or approval of the manuscript; and the decision to submit the manuscript for publication. The content is solely the responsibility of the authors and does not necessarily represent the official views of the NIH.

Competing interests statement: The authors have no conflicts of interest to disclose relevant to the subject matter in this manuscript.

For peer review only

For peer review only - http://bmjopen.bmj.com/site/about/guidelines.xhtml

INTRODUCTION

There are an estimated 4.2 million Americans with diabetic retinopathy, which is the most common cause of blindness in working-age U.S. adults.[1,2] The risk of severe vision loss decreases by 90% with early diagnosis and treatment, but fewer than half of the 29.1 million Americans with diabetes receive yearly recommended diabetic retinopathy screening.[3-5] Teleophthalmology is an evidence-based intervention proven to substantially improve diabetic eye screening rates and reduce blindness from diabetes.[6] A retinal camera is used to image patients' eyes in a convenient location, such as a primary care clinic (where more than 90% of patients with diabetes obtain their care).[7] These images are then electronically-transmitted to and evaluated by specialists at a distant site, typically within a timeframe of the same day to 1 week. Patients needing additional eye care are then identified for expedited treatment. The prevalence of diabetes, and the demand for eye screening, is projected to double by 2050 without a concurrent increase in the supply of eye care providers.[8] Thus, there is an urgent need to expand teleophthalmology use to improve screening rates and respond to growing demand.

In England, the National Health Service achieves screening rates of over 80% using teleophthalmology and subsequently, diabetic retinopathy is no longer the leading cause of blindness in working-age English adults.[6,9] Presently, successful implementation of teleophthalmology in the U.S. is largely limited to single-payer or highly specialized health systems.[10,11] Teleophthalmology programs in these settings have achieved sustained screening rates as high as 80% or more. Success in multi-payer settings has been much more limited. A multi-payer health system is one in which individuals (or their employers) pay for healthcare services through a variety of private or public health insurance sources, in contrast to a single-payer health system in which healthcare is paid for by a single payer (e.g. government-financed

For peer review only - http://bmjopen.bmj.com/site/about/guidelines.xhtml

healthcare supported by taxes).[12] A recent 5-year randomized-controlled trial in a multi-payer health system compared teleophthalmology to traditional screening methods (i.e. in-person dilated eye exams) and found initial improvement in screening rates with teleophthalmology, but screening rates declined within 18 months and did not exceed 55% even when teleophthalmology became available to both groups.[13] However, this study did not utilize a systematic implementation approach designed to sustain integration of this technology into the primary care workflow.

Teleophthalmology is particularly well suited to rural populations, which have less access and greater travel distances to obtain eye care.[14-18] Rural communities are largely served by multi-payer health systems, which are less likely to encourage preventive services because of poor reimbursement for such services due to insurers' financial incentives to focus on providing healthcare in the short-term.[12] Unfortunately, studies show that simply providing access to teleophthalmology in multi-payer health systems is insufficient to either achieve or sustain initial improvements in screening rates.[10,12,13,19,20] Patient and provider-related barriers to teleophthalmology have been postulated, but are poorly understood. These barriers may be magnified in rural populations because they are less insured, poorer, older, less likely to receive guideline-concordant care, and experience more chronic diseases than those in urban areas.[16-18,21]

We hypothesized that while teleophthalmology addresses some logistical barriers to diabetic eye screening, it may not address other important patient and provider barriers in multi-payer health systems. Qualitative research methods are used in health services research to explore complex phenomena needing further explanatory analysis, such as real-world patient and provider barriers to teleophthalmology use, through a rich description of key perspectives.[22] We conducted individual interviews to understand what prevents or motivates patients and primary care providers

For peer review only

For peer review only - http://bmjopen.bmj.com/site/about/guidelines.xhtml

(PCPs) to use teleophthalmology, as well as strategies to increase teleophthalmology use, in a rural multi-payer health system with an active teleophthalmology program.

RESEARCH DESIGN AND METHODS

Research Setting

We conducted standardized open-ended, individual interviews with patients with diabetes and PCPs at Mile Bluff Medical Center. Mile Bluff is a rural, multi-payer health system in Mauston, Wisconsin. A teleophthalmology program was established in 2015 (one year prior to the start of our study) in partnership with the University of Wisconsin-Madison. This program allows PCPs to refer patients for teleophthalmology with walk-in scheduling. Referrals are completed by PCPs in the Mile Bluff electronic health record (EHR). Retinal images are electronically-transmitted to and evaluated by University eye specialists. Imaging reports are then sent back to the PCP and patient within 1 week, which is consistent with the usual timeframe for receiving results of other clinical studies (e.g. lab tests and x-rays) provided by this rural health system and was considered acceptable to all patients and primary care providers in our study. Patients are charged $20 for the service and referred to local eye doctors for further care if found to have visually-significant eye disease.

This teleophthalmology program was established prior to our study based on the 2011 American Telemedicine Association Telehealth Practice Recommendations for Diabetic Retinopathy.[23] The Topcon NW400 camera (Topcon Medical Systems, Inc., Oakland, NJ, USA) was used to obtain a single 45-degree image of the disc and macula in each eye, along with an anterior segment photograph. If a fundus image was considered to be poor quality by the imager, then the camera's "small pupil" mode was used to capture additional images. If images remained

For peer review only

For peer review only - http://bmjopen.bmj.com/site/about/guidelines.xhtml

poor, then pharmacologic dilation using 0.5% tropicamide was performed with the patient's consent. The percentage of patients undergoing pharmacologic pupil dilation was 2.2% and the frequency of ungradable cases among all patients was 2.6%. Two years after the establishment of the teleophthalmology service, a quality improvement program outside the scope of this study was created to develop an implementation program to increase teleophthalmology utilization.

Interviews

We developed our interview guides using the *Chronic Care Model,* a framework for improving chronic disease management and guideline-concordant diabetes care.[24,25] Following a literature search on barriers and facilitators to teleophthalmology for diabetic eye screening, the patient interview guide (Supplemental Appendix S1) was tested and further refined with input from the University of Wisconsin Community Advisors on Research Design and Strategies (CARDS®), which is a group of lay community members trained to review patient research materials. The PCP interview guide (Supplemental Appendix S2) was tested and further refined with input from PCPs in the University of Wisconsin Primary Care Academics to Transform Healthcare (PATH). Members of the University of Wisconsin Institute for Clinical and Translational Research-Community Academic Partnership (UW ICTR-CAP) Qualitative Research Group also reviewed and provided feedback on both interview guides.

Standardized open-ended, individual interviews combined with flexible probes were conducted between July 2016 and April 2017 (1-2 years after the teleophthalmology program was established) to understand patient and PCP perspectives on teleophthalmology and diabetic eye screening. This approach to interviewing ensured that each interview covered all topics included in the guide consistently, but also allowed the interviewer latitude to explore specific participant

For peer review only - http://bmjopen.bmj.com/site/about/guidelines.xhtml

For peer review only

responses.[26] All interviews were conducted one-on-one by a female research specialist (R.S.) with training in qualitative research and certification as a nursing assistant. The interviewer did not have a relationship with the participants prior to the study and informed participants that she did not have specialized medical training in eye care. Patient interviews were conducted in-person (30-45 minutes) at a local library. PCP interviews were conducted over the phone (15-30 minutes) to accommodate their busy clinic schedules. Patients received $30 and PCPs received $50 compensation for their time. Interviews were audiotaped and transcribed verbatim with all personal identifiers redacted. The interviewer also took field notes during and after the interview.

Study Sample

The sample of 20 adult patients with diabetes (with and without experience with teleophthalmology) and 9 PCPs was drawn from patients and providers at Mile Bluff Medical Center. Adult patients (18 year or older) with a diagnosis of diabetes were recruited who either: (1) had teleophthalmology imaging within the preceding 2 months or (2) expressed interest in participating in a research study when previously contacted in a quality improvement telephone survey on diabetic eye screening. Fifty patients were invited to participate by a mailed letter and a follow-up phone call. All patients had a PCP from Mile Bluff Family or Internal Medicine. PCPs were recruited during a provider staff meeting with purposeful recruitment of providers having a range of training backgrounds reflective of their representation at Mile Bluff. No participants dropped out of the study. Sample sizes for both patients and PCPs were sufficient to reach informational redundancy in which no new information was obtained from additional interviews.[27]

Data Analysis

For peer review only - http://bmjopen.bmj.com/site/about/guidelines.xhtml

We began analyzing the interview transcripts using inductive analysis. Members of the research team (R.S.-research specialist and certified nursing assistant, N.Z.-research specialist with a Master of Public Health Degree, and Y.L.-clinical ophthalmologist and principal investigator) conducted independent open coding of the first 5 transcripts. Research team members then met with N.J.-Ph.D. qualitative methodologist to review these codes and agree upon an initial coding framework. A second coding cycle was then performed by 1 research team member (N.Z.) to fit codes into an evolving collection of higher-order categories. Consistency was ensured by the principal investigator (Y.L.) who dual-coded every 5th transcript. Throughout the analysis process, codes were iteratively reviewed by the entire research team, which met regularly to discuss and refine the first and second-order analytic categories pertinent to understanding facilitators and barriers to teleophthalmology use.

Research team discussions centered around the organization of themes into separate categories (e.g. lack of time) or grouping them within larger categories (e.g. logistical challenges), as well as how themes applied to patients, PCPs or to both groups. The team also used these meetings to explore other, less expected, themes emerging from the data. Unexpected findings included the influence of broader socio-ecological factors on rural residents' adherence with diabetic eye screening, which were beyond the scope of this study.[28] These included limited access to healthcare (e.g. long travel distances), anxiety stemming from family members' experiences with diabetes complications, and the daily burden of diabetes management. Additionally, implementation strategies to increase teleophthalmology use were deductively coded and categorized using the *Chronic Care Model* as a framework for directed content analysis. Members of the UW ICTR-CAP Qualitative Research Group also reviewed parts of the interview data and

For peer review only - http://bmjopen.bmj.com/site/about/guidelines.xhtml

coding methods. We used NVivo software, Version 11.4.1 (QSR International, Melbourne, Australia) for data management.

Our data analysis was validated using member checking with a subset of interview participants (n=9 patients, n=6 PCPs) at two separate patient and provider stakeholder group meetings organized as part of a quality improvement initiative to increase diabetic eye screening at Mile Bluff that began 2 years after the establishment of the teleophthalmology program to increase its utilization.[29] Participating patients and providers judged our interpretation of the interview data to be accurate and complete. Furthermore, the most important or "top" barriers and facilitators were identified through the Nominal Group Technique, in which each participant sequentially shares one additional idea with the group (until no new ideas are generated) and then all participants anonymously cast their written votes.[30] Our report of this study followed the Consolidated criteria for reporting qualitative research (COREQ).[31]

Patient Involvement

Patients from the University of Wisconsin Community Advisors on Research Design and Strategies (CARDS®) were involved in the design of the study by providing feedback on development of the patient interview guide. In addition, a patient stakeholder group (Mile Bluff Diabetes Patient Advisory Council) was established among participants in this study to provide member-checking of our results and advise on dissemination of the results.

Ethics/Institutional Review Board Approval

The University of Wisconsin School of Medicine and Public Health Human Subjects Institutional Review Board (IRB) staff reviewed all study activities in detail and determined that

For peer review only - http://bmjopen.bmj.com/site/about/guidelines.xhtml

For peer review only

this research met criteria for exemption from full IRB review based on U.S. federal Common Rule (45 CFR 46.101(b)), which provides exemptions for interview research protocols with minimal risk to participants. Verbal consent was obtained from all study participants.

RESULTS

Patient and Provider Characteristics

All patients were Caucasian adults diagnosed with type-2 diabetes (Table 1). Fifty percent of patients had experience with teleophthalmology and most (85%) self-reported high health literacy in response to the Single Item Literacy Screener.[32] Only one patient among the 20 patient participants underwent pupil dilation. There were no significant differences in the responses of this patient compared to those of the other 19 patients who did not undergo pupil dilation. PCPs were predominantly male (77.8%) and had a variety of training backgrounds (Table 1). Most had been in practice for over 10 years (77.8%) and had referred patients for teleophthalmology (87.5%).

Table 1. Patient and Primary Care Provider Demographics

Participant Characteristics	Median or Percentage
Patients (n=20)	
Age	67 years (range: 46-86 years)
Male	55%
Ethnicity (self-reported)	
Caucasian, non-Hispanic	100%
Diagnosis of Type-2 Diabetes	100%
Had Teleophthalmology Screening	50%
Duration of diabetes	
<5 years	40%
5-19 years	30%
20+ years	30%
Highest Level of Education	
College graduate	10%

For peer review only - http://bmjopen.bmj.com/site/about/guidelines.xhtml

Some college/tech school	30%
High school graduate or GED	35%
Some high school	15%
Grade 8 or less	10%
Health literacy (Single Item Literacy Screener)	
High	85%
Moderate	10%
Low	5%
Primary Care Providers (n=9)	
Male	77.8%
Training Background	
Physician (MD/DO)	44.4%
Physician Assistant (PA-C)	33.3%
Nurse Practitioner (DNP)	11.1%
Nurse (RN)	11.1%
Years in Practice	
>10 years	77.8%
5-10 years	0%
0-5 years	22.2%
Have Referred Patients for Teleophthalmology	87.5%

Patient Barriers and Facilitators

Major patient barriers to teleophthalmology use included being unfamiliar with teleophthalmology, misconceptions about diabetic eye screening, and logistical challenges (Table 2).

Table 2. Patient and Primary Care Provider Barriers and Facilitators

Patients	Barriers	- Unfamiliar with teleophthalmology*<br>- Misconceptions about diabetic eye screening*<br>- Logistical challenges* (e.g. time, transportation, out-of-pocket cost)<br>- Eye problems requiring in-person exam (e.g. glasses or glaucoma)<br>- Anxiety about receiving bad news regarding their eyes
	Facilitators	- Recommendation from primary care provider*<br>- Convenience of teleophthalmology* (e.g. same-day scheduling, location, quick)

For peer review only - http://bmjopen.bmj.com/site/about/guidelines.xhtml

		- Belief that diabetic eye screening is important for preventing vision loss<br>- Knowing that pupil dilation is usually not necessary<br>Teleophthalmology is considered a high-quality service due to University affiliation
Primary Care Providers	Barriers	- Difficulty identifying when patients are due for diabetic eye screening*<br>- Unfamiliar with teleophthalmology*<br>- Time constraints (e.g. many competing tasks during clinic visit)<br>- Concerns about conflicts with local eye doctors<br>- Concerns about patients' barriers (e.g. out-of-pocket cost)
	Facilitators	- Ease of referral process and results communication*<br>- Perceived benefits to patients (e.g. convenience, cost)<br>- Improved patient adherence with diabetic eye screening<br>- Benefits to the healthcare organization (e.g. increased reimbursement for improved quality metrics)

*Top barrier or facilitator identified at patient or primary care provider stakeholder meeting

For peer review only

Major patient facilitators included a recommendation from the patient's PCP and factors related to convenience. Most patients were unfamiliar with teleophthalmology, which prevented them from taking the initiative to seek a teleophthalmology referral from their PCP for diabetic eye screening. In addition, several patients demonstrated a limited understanding of diabetic eye disease and the importance of screening.

> "I don't see an advantage to getting [my eyes] checked every year, unless you are having issues…" (Patient 5)

Many patients expressed the belief that having "good" vision and the absence of visual symptoms indicated that they do not have diabetic eye disease. The advantage of identifying and treating the disease at earlier, asymptomatic stages was not well understood. Patients were often unaware that ongoing screening was needed because the risk of retinopathy increases over time. Some patients

For peer review only - http://bmjopen.bmj.com/site/about/guidelines.xhtml

believed that having one diabetic eye screening that was negative, even if it was several years ago, was sufficient and that additional screening was unnecessary.

Interestingly, neither a detailed understanding of diabetic eye disease nor the purpose of screening was necessary for patient adherence if the patient believed that yearly diabetic eye screening was important. This belief was often attributed to a strong recommendation from their PCP, which was the most common reason patients reported for using teleophthalmology.

> "My doctor thinks [teleophthalmology], you know, is a good test. And for me that's pretty much all I need." (Patient 16)

A PCP's recommendation was a major patient motivator because of the high level of trust patients placed in their PCP. Patients reported that they obtained most of their health information directly from their PCP as well as through the clinic's diabetes education programs and informational handouts. Some patients also described maintaining independence, continuing to enjoy hobbies, and caring for other family members as important reasons for protecting their vision through screening. Their PCP's recommendation to pursue teleophthalmology screening reinforced these personal values.

Teleophthalmology was frequently endorsed as being "quick, easy, and painless" (Patient 5). When comparing teleophthalmology to traditional, in-person eye exams, many patients appreciated that teleophthalmology was conveniently-located in the same building as their primary care provider and accommodated walk-in scheduling with short wait times. In addition, they described the imaging as highly efficient, often taking "less than 5 minutes" (Patient 19). Teleophthalmology was often preferred by patients because it was more comfortable than

For peer review only - http://bmjopen.bmj.com/site/about/guidelines.xhtml

For peer review only

traditional eye exams since pharmacologic pupil dilation was usually not needed. Pharmacologic pupil dilation was reported as a significant barrier to obtaining traditional eye exams.

> "Look at it from my viewpoint. I'm upstairs at the doctor's office and he says, 'Maybe you should go get [an eye photo] done…' You walk downstairs… sit down for 10 minutes… you get it done and you go home… I mean, it's so simple." (Patient 17)

> "The last thing I want to do is lose my eyesight [from diabetes] and before [teleophthalmology], I wasn't big on going to the eye doctor…" (Patient 16)

> "I have a terrible time when they dilate my eyes. The light, it hurts so bad." (Patient 5)

Although teleophthalmology addresses many barriers to diabetic eye screening, several important challenges remain for patients in this older, rural population with diabetes, many of whom live on a limited income. Teleophthalmology is only available in one primary care clinic so patients at other clinic locations were required to travel from their regular clinic to use it (median travel distance: 13.8 miles, range: 9.2-23.1 miles). Most patients also reported the need to pay out-of-pocket as a barrier due to limited insurance coverage for teleophthalmology.

PCP Barriers and Facilitators

Major PCP barriers to teleophthalmology use included difficulties identifying when patients are due for diabetic eye screening and being unfamiliar with teleophthalmology, while major facilitators were the ease of the referral process and results communication (Table 2). PCPs reported that they often did not have access to patients' eye records since all eye care providers in

For peer review only - http://bmjopen.bmj.com/site/about/guidelines.xhtml

this community practice outside their health system and utilize different electronic health records (EHRs). Thus, PCPs depend on eye care providers to send diabetic eye screening documentation, which is not consistently performed. PCPs reported insufficient reminders, time, and resources to “track down” eye records as well as to discuss and refer patients for teleophthalmology as significant barriers. For example, enlisting the help of a medical assistant to request records from the patient’s eye doctor was difficult due to time constraints during a typical PCP appointment. When a PCP cannot easily find these records, they would usually rely on the patient’s self-reported date of their last diabetic eye screening, which has limited accuracy.

> “I don’t have an easy way in my electronic records to see if [patients] had [diabetic eye screening] done and the patients never remember exactly when [it was done] ...” (PCP 7)

Faced with so many competing demands, PCPs often prioritized more urgent medical issues over teleophthalmology.

> “[As a provider] you are covering so many things… the foot exam, checking their cholesterol… [patients say] they saw the eye doctor and then you just go on to something else…” (PCP 4)

PCPs also reported being unfamiliar with teleophthalmology, which made some hesitant to refer patients. Some PCPs also worried about potential conflicts with local eye doctors and preferred to refer patients to eye doctors whom PCPs felt would be better suited to judge whether teleophthalmology would be appropriate.

For peer review only - http://bmjopen.bmj.com/site/about/guidelines.xhtml

> "I guess I just don't want to refer [a patient] in error if it's not really what the service is meant for." (PCP 3)

> "[I]… tend to refer patients to the ophthalmologists and they would probably be the ones to decide whether they were… teleophthalmology candidates" (PCP 5).

The most important facilitators for PCPs were the ease of referral process and results communication.

> "[Teleophthalmology] was probably one of the easiest referrals I've done, and the turnaround [time for receiving results] was by far the best... I've had" (PCP 3).

Not only did PCPs describe teleophthalmology as being convenient for their patients, but also endorsed it for making it easier for PCPs to document referrals and reliably receive reports in their EHR among patients who had received diabetic eye screening. Primary care providers were also motivated to use teleophthalmology because they believed that this technology made it easier and more likely for patients to obtain diabetic eye screening. If patients did not have access to teleophthalmology, patients would otherwise need to make their own appointments with an eye care provider for a dilated eye exam to obtain diabetic eye screening, which was more difficult due to the multiple patient barriers we described.

A Model to Understand Patient and PCP Barriers in the Teleophthalmology Referral Process

For peer review only - http://bmjopen.bmj.com/site/about/guidelines.xhtml

Based on our results, we developed a model for understanding where key patient and PCP barriers occur during the primary care teleophthalmology referral process (Figure 1). This model illustrates the temporal relationship between barriers in the teleophthalmology referral process, which is important for understanding the relative impact and mapping of possible strategies to overcome barriers in this process. PCPs are expected to initiate the referral process, but have difficulty remembering to ask patients about diabetic eye screening and identifying when patients are due. Next, they may have limited time to generate the referral and explain its purpose to the patient, especially when both the patient and the PCP are less familiar with teleophthalmology. Finally, referred patients may choose not to participate in teleophthalmology due to being unfamiliar with the technology, misconceptions or a lack of understanding about the importance of diabetic eye screening, and/or logistical difficulties related to time, travel or cost. This figure helps to demonstrate why strategies aimed at the provider and health system may be more effective in increasing teleophthalmology use because of the earlier role of the primary care provider and the later role of the patient in completing the referral process

Possible Implementation Strategies

Patients and PCPs described possible implementation strategies to increase teleophthalmology use, which were categorized using the *Chronic Care Model* (Table 3). A variety of system-based implementation strategies were identified, primarily targeting the health system by streamlining PCP and clinic staff workflow processes (Table 3).

Table 3. Strategies to Increase Teleophthalmology Use Mapped to Chronic Care Model (CCM)

For peer review only - http://bmjopen.bmj.com/site/about/guidelines.xhtml

CCM Component	Target	Examples of Strategies
Health System Organization & Delivery System Design	Health System, PCPs, and Clinic Staff	- Workflow changes including clinic staff checklists and delegation of referrals† - Provider/staff training to increase familiarity with teleophthalmology† - Convenient scheduling and location† - Provide financial incentives to individual PCPs for diabetic eye screening performance
Decision Support & Clinical Information Systems	Health System, PCPs, and Clinic Staff	- Best practice alert in EHR when patient is due for diabetic eye screening† - Streamline processes for getting diabetic eye screening documentation into EHR† - Provide PCPs with feedback/data on diabetic eye screening performance (e.g. quarterly) - Generate lists of patients due for diabetic eye screening for clinic staff to contact
Self-Management Support & Community Resources	Patients, Families, Community Members, and Clinic Staff	- Patient education materials provided at primary care clinic visits† - Increase education about diabetic eye screening in diabetes self-management classes† - Publicize teleophthalmology services† (e.g. local media ad community health fairs) - PCP clinic staff facilitate diabetic eye screening by calling patients and sending letters when due

†Strategies that can address top barriers or facilitators from Table 2

Strategies that addressed top barriers or facilitators included best practice alerts in the EHR to remind PCPs when patients are due for diabetic eye screening, improving PCP access to diabetic eye screening records, and clinic workflow changes such as the delegation of teleophthalmology referrals to clinic staff. In contrast, fewer patient-directed strategies were identified. Arranging the scheduling and location for teleophthalmology imaging to maximize patient convenience was emphasized. Suggested solutions included having teleophthalmology available during weekend or evening hours, as well as obtaining a camera for each primary care clinic or rotating the current camera between primary care clinics (e.g. a “mobile” camera approach). Many PCPs recommended providing further training to both providers and clinic staff on the use and purpose of teleophthalmology. Patients reported that greater education about the importance of diabetic eye

For peer review only - http://bmjopen.bmj.com/site/about/guidelines.xhtml

screening, particularly to address common misconceptions, and increasing community awareness of teleophthalmology services would increase their likelihood of using teleophthalmology. Many patients preferred receiving written patient education materials from their PCP clinic or through presentations in diabetes education classes. They also recommended publicizing teleophthalmology through local newspaper or television advertisements.

DISCUSSION

Teleophthalmology is effective for increasing diabetic eye screening rates in rural populations.[14-18] However, there may be poor early adoption of this technology or a lack of sustained use by patients and PCPs despite having both equipment and trained personnel readily available—and even when the service is provided at no cost to the patient.[13] In this qualitative study, we identified patient and provider barriers, facilitators, and implementation strategies to improve and sustain teleophthalmology use over time for diabetic eye screening in a rural, multi-payer primary care setting.

Top patient barriers include being unfamiliar with teleophthalmology, misconceptions about diabetic eye screening, and logistical challenges, which were consistent with prior studies examining patient barriers to teleophthalmology and traditional methods for diabetic eye screening.[33-41] Patients reported that the most common reason they obtained screening was a strong recommendation from their PCP. Many prior studies that did not ask about provider recommendation have found that patients were primarily motivated to seek diabetic eye screening to prevent vision loss,[33,34,36] but this was less frequently reported by patients in our study. We found that while many patients have limited knowledge and understanding of the purpose of diabetic eye screening, they still obtained teleophthalmology screening when recommended by

For peer review only - http://bmjopen.bmj.com/site/about/guidelines.xhtml

their PCP. Our data agrees with literature showing that patients report provider recommendation as the strongest motivator of obtaining preventative screenings.[40,42] A survey of nearly 2,000 patients with diabetes found provider recommendation to be the most strongly-associated with patient adherence with diabetic eye screening (odds ratio 341, 95% confidence interval 164 to 715), and was much more strongly associated with adherence than patient knowledge about the effects of diabetic retinopathy on vision (odds ratio 3.3, 95% confidence interval 2.0 to 5.5).[40] We believe this is due to patients' high level of trust in their providers, which has been linked to increased adherence with diabetic eye screening among rural older adults.[43]

In addition, our model demonstrates that the PCP initiates the teleophthalmology referral and that the patient's role in completing diabetic eye screening is often further downstream. The organization of the clinic requires that the primary care provider provide a referral for a patient to obtain teleophthalmology. As a result of this temporal relationship, if the PCP is not reminded to consider making the referral, the patient may not obtain teleophthalmology. Some strategies identified to address this barrier are the delegation of the teleophthalmology referrals to clinic staff such as medical assistants or allowing patient self-referral. Some PCPs reported concerns about the potential conflict between providing teleophthalmology in primary care and reduced referrals to local eye doctors, but studies have shown that teleophthalmology actually increases patient utilization of local eye care services by bringing into care many patients who would not otherwise obtain yearly eye exams.[44,45] While patient education is important, our model suggests that implementation strategies to address PCP barriers may be more effective for increasing teleophthalmology use because of the PCP's primary role in the referral process. This is supported by studies that showed no significant differences in demographics or beliefs among patients who are or are not adherent with diabetic eye screening.[36] Instead, our data suggest that PCPs and clinic

For peer review only - http://bmjopen.bmj.com/site/about/guidelines.xhtml

staff characteristics may be more influential in determining whether patients adhere with screening.

System-level implementation strategies are needed to provide PCPs with the information needed to make appropriate referrals, including better communication with eye care providers (e.g. up-to-date documentation of diabetic eye exams) and other EHR enhancements (e.g. systematic reminders when patients are due for screening and streamlining the referral process).[33,35] We recognize that the cost and availability of certain EHR functionalities are a potential limitation for some health systems and that lower-cost alternatives or other implementation strategies may be more important in settings with limited resources. For example, one of the medical assistants in this primary care clinic devised a low-cost system of flagging paper charts with a color-coded diabetes checklist to make it easier for the PCP identify patients due for diabetic eye screening. Addressing logistical barriers for patients by providing same-day, walk-in scheduling at a convenient location and minimizing out-of-pocket costs are also important. In addition, PCP, clinic staff, and patient education as well as publicizing teleophthalmology throughout the community were also recommended by interview participants. Of note, we did not ask study participants to differentiate between strategies they believed would increase initial adoption or sustain use of teleophthalmology over time. Determining which of the strategies they identified would be useful for one or both purposes should be evaluated in future studies.

There are several limitations of our study. All patients reported having experience with diabetic eye screening and may have been more willing to participate than patients who do not follow diabetes eye screening guidelines. However, studies show that similar barriers exist for patients who do and those who do not adhere with screening guidelines.[36] Our teleophthalmology program requires PCP referral and different barriers may occur in teleophthalmology programs

For peer review only - http://bmjopen.bmj.com/site/about/guidelines.xhtml

allowing patient self-referral. The majority of patients in our study reported high levels of general health literacy (85%), which was greater than that reported by a similar population of rural adults (70.9%).[46] We did not systematically assess knowledge of diabetic eye screening from patients in our study because prior literature has demonstrated that more than half of patients with diabetes are aware of screening guidelines and patient knowledge of guidelines is insufficient to ensure adherence with diabetic eye screening guidelines.[35,47]

In addition, all patients were Caucasian and native English speakers, which reflected the composition of this community. A study of predominantly Latino and African-American patients in an urban, safety-net clinic found gaps in patient knowledge of diabetic retinopathy and screening, which prevented them from using teleophthalmology.[41] However, that study did not examine other factors that may limit urban patients' utilization of teleophthalmology. The rural population in our study faces limited access to care, which is shared by many non-Caucasian populations, including those in underserved urban areas and in low- to medium-income countries. While we expect some of the implementation strategies we identified to translate to these populations, tailoring of strategies to the local community may be important to account for differences in cultural backgrounds and available healthcare resources. Further studies among patients from urban, other ethnic groups, and non-native English-speaking backgrounds are needed to assess the generalizability of our findings.

Our study is one of the first to directly identify implementation strategies suggested by patients and PCPs that address their barriers and facilitators to using teleophthalmology for diabetic eye screening. In this rural, multi-payer health system, PCPs initiate teleophthalmology referrals and patients reported that a strong recommendation from their PCP was the most important motivator for obtaining screening. Thus, system-level implementation strategies

For peer review only - http://bmjopen.bmj.com/site/about/guidelines.xhtml

focusing on the PCP and clinic staff workflow processes appear to have the greatest potential to increase and maintain utilization in this setting. Future studies testing these strategies in multiple rural and urban health systems can evaluate their relative impact and the generalizability of our findings on increasing teleophthalmology use and diabetic eye screening rates. Furthermore, interviews with health system administrators may be useful for identifying additional barriers to establishing and sustaining teleophthalmology programs. Implications for other clinics include the importance of strategies targeting health system workflow processes along with educating patients, providers, and staff. Clinics may benefit from assessing their own unique patient and provider barriers and facilitators, as well as their available resources, in order to tailor the selection of effective strategies to increase teleophthalmology utilization.

CONCLUSIONS

Teleophthalmology can substantially increase diabetic eye screening rates and prevent blindness. While this technology addresses numerous logistical barriers to diabetic eye screening, we found several additional barriers to its use by patients and PCPs. System-based implementation strategies primarily targeting PCP barriers in conjunction with improved patient and provider education may increase teleophthalmology use in rural, multi-payer primary care clinics.

For peer review only - http://bmjopen.bmj.com/site/about/guidelines.xhtml

ACKNOWLEDGEMENTS

The authors acknowledge the UW ICTR-CAP Qualitative Research Group (QRG), Wisconsin Network for Research Support (WINRS), Community Advisors on Research Design and Strategies (CARDS)®, and the Primary Care Academics Transforming Healthcare (PATH) Writing Collaborative for their feedback on interview guides and recruitment methodology. We thank the Mile Bluff Medical Center Diabetes Patient Advisory Council and Diabetes Quality Care Team for providing feedback to refine our research results.

Prior abstract publication: This study was presented at the 2017 Association for Research in Vision and Ophthalmology (ARVO) Annual Meeting in Baltimore, MD, USA.

Author contributions: Y.L., R.S., and N.J. developed the interview guides. R.S. conducted the interviews. Y.L., N.Z., J.C., and N.J. analyzed the data. J.M., R.K., T.B., and M.S. contributed to the discussion and reviewed/edited the manuscript. Y.L. and N.Z. wrote the manuscript.

Guarantor statement: Dr. Liu accepts full responsibility for this work as a whole, including the study design, access to the data, and the decision to submit and publish the manuscript.

Data sharing statement: Data from this study are available upon request from the corresponding author to those who fulfill requirements set by the University of Wisconsin Institutional Review Board.

For peer review only - http://bmjopen.bmj.com/site/about/guidelines.xhtml

Figure Legends (in order of appearance in the text)

Table 1. Patient and Primary Care Provider Demographics

Table 2. Patient and Primary Care Provider Barriers and Facilitators

Figure 1. Barriers in the Teleophthalmology Referral Process

Table 3. Strategies to Increase Teleophthalmology Use Mapped to Chronic Care Model (CCM)

For peer review only

For peer review only - http://bmjopen.bmj.com/site/about/guidelines.xhtml

REFERENCES

1. National diabetes statistics report: estimates of diabetes and its burden in the United States, 2014. *Atlanta, GA: US Department of Health and Human Services* 2014;2014

2. National Eye Institute Factsheet, 2015. National Institutes of Health, 2015. Available from https://nei.nih.gov/sites/default/files/nei-pdfs/NEI_Factsheet_July_2015_v2.pdf

3. Early Treatment Diabetic Retinopathy Study Research Group. Early photocoagulation for diabetic retinopathy: ETDRS report number 9. *Ophthalmology* 1991;98(5):766-85.

4. Lee PP, Feldman ZW, Ostermann J, et al. Longitudinal rates of annual eye examinations of persons with diabetes and chronic eye diseases. *Ophthalmology* 2003;110(10):1952-59.

5. Centers for Disease Control and Prevention. National diabetes fact sheet: national estimates and general information on diabetes and prediabetes in the United States, 2010. *http://www caldiabetes org/content_display cfm? FontSize= normal&contentID= 562&CategoriesID= 31* 2011

6. Scanlon PH. The English National Screening Programme for diabetic retinopathy 2003-2016. *Acta Diabetologica* 2017;54(6):515-25. doi: 10.1007/s00592-017-0974-1

7. Davidson JA. The Increasing Role of Primary Care Physicians in Caring for Patients With Type 2 Diabetes Mellitus. *Mayo Clinic Proceedings* 2010;85(12 Suppl):S3-S4. doi: 10.4065/mcp.2010.0466

8. Boyle JP, Thompson TJ, Gregg EW, et al. Projection of the year 2050 burden of diabetes in the US adult population: dynamic modeling of incidence, mortality, and prediabetes prevalence. *Population health metrics* 2010;8(1):29.

9. Liew G, Michaelides M, Bunce C. A comparison of the causes of blindness certifications in England and Wales in working age adults (16–64 years), 1999–2000 with 2009–2010. *BMJ open* 2014;4(2):e004015.

10. Garg S, Zimmer-Galler I. Retinal Physician - Telemedicine and Remote Imaging for Diabetic Retinopathy Evaluation: An Update. *Retinal Physician* 2013;10(March 2013):49-51.

11. Whited JD, Datta SK, Aiello LM, et al. A modeled economic analysis of a digital teleophthalmology system as used by three federal healthcare agencies for detecting proliferative diabetic retinopathy. *Telemedicine Journal & E-Health* 2005;11(6):641-51.

12. Hussey P, Anderson GF. A comparison of single-and multi-payer health insurance systems and options for reform. *Health policy* 2003;66(3):215-28.

For peer review only - http://bmjopen.bmj.com/site/about/guidelines.xhtml

13. Mansberger SL, Sheppler C, Barker G, et al. Long-term comparative effectiveness of telemedicine in providing diabetic retinopathy screening examinations: a randomized clinical trial. *JAMA ophthalmology* 2015;133(5):518-25.

14. Ahmed MD, Petrany SM, Fry II, et al. Screening diabetic and hypertensive patients for ocular pathology using telemedicine technology in rural West Virginia: a retrospective chart review. *West Virginia Medical Journal* 2013;109(1):6-10.

15. Cummings DM, Morrissey S, Barondes MJ, et al. Screening for diabetic retinopathy in rural areas: the potential of telemedicine. *The Journal of Rural Health* 2001;17(1):25-31.

16. Dansky KH, Dirani R. The use of health care services by people with diabetes in rural areas. *The Journal of Rural Health* 1998;14(2):129-37.

17. Krishna S, Gillespie KN, McBride TM. Diabetes burden and access to preventive care in the rural United States. *J Rural Health* 2010;26(1):3-11. doi: 10.1111/j.1748-0361.2009.00259.x

18. Leese GP, Ahmed S, Newton RW, et al. Use of mobile screening unit for diabetic retinopathy in rural and urban areas. *BMJ* 1993;306(6871):187-89.

19. Arora S, Kurji AK, Tennant MTS. Dismantling sociocultural barriers to eye care with tele-ophthalmology: lessons from an Alberta Cree community. *Clinical & Investigative Medicine* 2013;36(2):57-63.

20. Chedid EH, Golden QR, Jager RD. Operational challenges in delivery of a charity care program for diabetic retinopathy screening in an urban setting. *The Permanente Journal* 2013;17(1):21.

21. American Diabetes Association. 10. Microvascular Complications and Foot Care. *Diabetes Care* 2017;40(Suppl 1):S88-S98. doi: 10.2337/dc17-S013

22. Sofaer S. Qualitative methods: what are they and why use them? *Health services research* 1999;34(5 Pt 2):1101.

23. Li HK, Horton M, Bursell S-E, et al. Telehealth practice recommendations for diabetic retinopathy. *Telemedicine and e-health* 2011;17(10):814-37.

24. Bodenheimer T, Wagner EH, Grumbach K. Improving Primary Care for Patients With Chronic Illness: The Chronic Care Model, Part 2. *JAMA* 2002;288(15):1909-14.

25. Bodenheimer T, Wagner EH, Grumbach K. Improving primary care for patients with chronic illness: the chronic care model, Part 2. *JAMA* 2002;288(15):1909-14.

26. Patton MQ. Qualitative research and evaluation methods. Third ed: Sage Publications, Inc. 2002.

For peer review only - http://bmjopen.bmj.com/site/about/guidelines.xhtml

27. Sandelowski M. Sample size in qualitative research. *Research in Nursing & Health* 1995;18(2):179-83. doi: 10.1002/nur.4770180211

28. Liu Y, Zupan NJ, Shiyanbola OO, et al. Factors influencing patient adherence with diabetic eye screening in rural communities: A qualitative study. *PLoS One* 2018;13(11):e0206742. doi: 10.1371/journal.pone.0206742

29. Birt L, Scott S, Cavers D, et al. Member checking: A tool to enhance trustworthiness or merely a nod to validation? *Qualitative Health Research* 2016;26(13):1802-11.

30. Delbecq AL, Van de Ven AH, Gustafson DH. Group techniques for program planning: A guide to nominal group and Delphi processes: Scott Foresman 1975.

31. Tong A, Sainsbury P, Craig J. Consolidated criteria for reporting qualitative research (COREQ): a 32-item checklist for interviews and focus groups. *Int J Qual Health Care* 2007;19(6):349-57. doi: 10.1093/intqhc/mzm042

32. Morris NS, MacLean CD, Chew LD, et al. The Single Item Literacy Screener: evaluation of a brief instrument to identify limited reading ability. *BMC Fam Pract* 2006;7:21. doi: 10.1186/1471-2296-7-21

33. Ellish NJ, Royak-Schaler R, Passmore SR, et al. Knowledge, attitudes, and beliefs about dilated eye examinations among African-Americans. *Invest Ophthalmol Vis Sci* 2007;48(5):1989-94. doi: 10.1167/iovs.06-0934

34. Glasson NM, Larkins SL, Crossland LJ. What do patients with diabetes and providers think of an innovative Australian model of remote diabetic retinopathy screening? A qualitative study. *BMC Health Services Research* 2017;17:158.

35. Hartnett ME, Key IJ, Loyacano NM, et al. Perceived barriers to diabetic eye care: qualitative study of patients and physicians. *Arch Ophthalmol* 2005;123(3):387-91. doi: 10.1001/archopht.123.3.387

36. Hipwell AE, Sturt J, Lindenmeyer A, et al. Attitudes, access and anguish: a qualitative interview study of staff and patients' experiences of diabetic retinopathy screening. *BMJ open* 2014;4(12):e005498.

37. Holley CD, Lee PP. Primary care provider views of the current referral-to-eye-care process: focus group results. *Invest Ophthalmol Vis Sci* 2010;51(4):1866-72. doi: 10.1167/iovs.09-4512

38. Lewis K, Patel D, Yorston D, et al. A Qualitative Study in the United Kingdom of Factors Influencing Attendance by Patients with Diabetes at Ophthalmic Outpatient Clinics. *Ophthalmic Epidemiology* 2007;14(6):375-80.

39. Lindenmeyer A, Sturt JA, Hipwell A, et al. Influence of primary care practices on patients' uptake of diabetic retinopathy screening: a qualitative case study. *Br J Gen Pract* 2014;64(625):e484-92. doi: 10.3399/bjgp14X680965

For peer review only

For peer review only - http://bmjopen.bmj.com/site/about/guidelines.xhtml

40. van Eijk KND, Blom JW, Gussekloo J, et al. Diabetic retinopathy screening in patients with diabetes mellitus in primary care: Incentives and barriers to screening attendance. *Diabetes Research and Clinical Practice* 2012;96(1):10-16.

41. George SM, Hayes EM, Fish A, et al. Understanding the Knowledge Gap Experienced by U.S. Safety Net Patients in Teleretinal Screening. *AMIA Annual Symposium Proceedings* 2017;2016:590-99.

42. Guerra CE, Schwartz JS, Armstrong K, et al. Barriers of and facilitators to physician recommendation of colorectal cancer screening. *J Gen Intern Med* 2007;22(12):1681-8. doi: 10.1007/s11606-007-0396-9

43. Bell RA, Arcury TA, Ip E, et al. Correlates of physician trust among rural older adults with diabetes. *Am J Health Behav* 2013;37(5):660-6. doi: 10.5993/AJHB.37.5.10

44. Kirkizlar E, Serban N, Sisson JA, et al. Evaluation of Telemedicine for Screening of Diabetic Retinopathy in the Veterans Health Administration. *Ophthalmology* 2013;120(12):2604-10. doi: 10.1016/j.ophtha.2013.06.029

45. Owsley C, McGwin G, Lee DJ, et al. Diabetes eye screening in urban settings serving minority populations: detection of diabetic retinopathy and other ocular findings using telemedicine. *JAMA ophthalmology* 2015;133(2):174-81.

46. Zahnd WE, Scaife SL, Francis ML. Health Literacy Skills in Rural and Urban Populations. *American Journal of Health Behavior* 2009;33(5):550-57. doi: 10.5993/ajhb.33.5.8

47. Weiss DM, Casten RJ, Leiby BE, et al. Effect of Behavioral Intervention on Dilated Fundus Examination Rates in Older African American Individuals With Diabetes Mellitus: A Randomized Clinical Trial. *JAMA Ophthalmol* 2015;133(9):1005-12. doi: 10.1001/jamaophthalmol.2015.1760

For peer review only

For peer review only - http://bmjopen.bmj.com/site/about/guidelines.xhtml

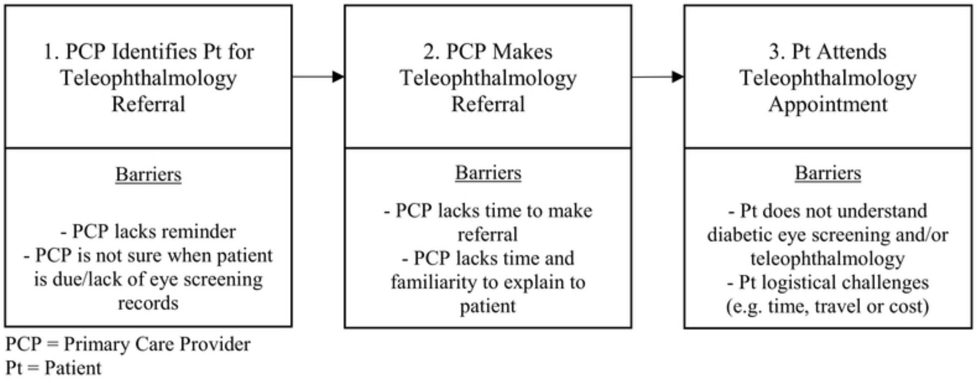

Figure 1. Barriers in the Teleophthalmology Referral Process

68x27mm (300 x 300 DPI)

Supplemental Appendix S1. Patient Interview Guide

Introduction:

Thank you so much for taking the time to meet with me. Our research team at the University of Wisconsin is working with the Mile Bluff Medical Center to learn more about patient experiences with diabetic eye exams.

You were invited to participate because you are a Mile Bluff Medical Center patient who has diabetes. I expect our conversation will last about 45 minutes. At the end of our discussion, I have a few questions about your background. Participation in this interview is voluntary. You can stop the interview at any time and if there are any questions you don't want to answer you can just tell me to skip those. Everything you tell me today will be kept confidential. Only our research team led by Dr. Yao Liu, a UW eye doctor, will have access to this information.

I will be audio recording this interview so that I can review our discussion later and make sure I accurately get all the ideas and opinions that you share. This interview is about <u>your</u> personal experience with eye exams. There are no "right" or "wrong" answers.

Do you have any questions before we begin? If you think of any questions as we go along, feel free to ask them any time.

Part I. Interview Questions

First, I'd like to ask you some general questions about how you take care of your diabetes.

What are things you do each day that are related to your diabetes?

Prompts:

- ☐ There are many things people do to take care of their diabetes. What do you see as the biggest priorities for you?
- ☐ *[if none]* What are some of the things your doctor may have discussed with you that may be important for people with diabetes to do?
- ☐ Are there things that you watch out for that tell you your diabetes may be getting worse?

Now I'd like to show you some pictures. These photos show two ways of doing eye checks for people with diabetes. The top photo shows a Traditional Eye Exam, where an eye doctor uses eye drops to dilate and examine your eyes. The bottom photo shows an Eye Photo Test, where a technician uses a special camera to take photos of your eyes.

Some people get yearly eye checks as part of their diabetes care. What do you think are the advantages of this? What are possible disadvantages to getting these eye checks?

Prompts:

- ☐ How do you know when it is time for you to get an eye check for diabetes?
- ☐ Why did you choose to get your eyes checked?

In your experience, how easy is it to regularly get diabetic eye checks as compared to *[use their example about something they do for their diabetes]*?

Prompts:

- ☐ Can you tell me more about why you find it easier/harder?

For peer review only

- ☐ You mentioned *[x]* as one thing that makes it hard to regularly get eye exams. Can you tell me more about that? What else might make it hard?
- ☐ What do you think might make it easier for you or other people with diabetes in your community to regularly get eye exams?

How do you find information about diabetic eye checks?

Prompts:

- ☐ From where do you get this information?
- ☐ Are there helpful resources here in your community?
- ☐ What support or resources do you think would help people learn more about this or help them get their eyes checked?
- ☐ *[If they don't currently get information about eye exams.]* Where do you get information about what you need to do related to diabetes?

[If patient has not had teleophthalmology, jump to page 4: Version II. Clinical Eye Exam]

Version I. Teleophthalmology

I believe you had the Eye Photo Test *[point to photo]* ***[x]*** **weeks/months ago, is this correct?**

Tell me about your experience starting with how you first learned about the Eye Photo Test.

Prompts:

- ☐ Do you remember who explained this test to you?
- ☐ What did they tell you about it?
- ☐ Did you get all the information you wanted?
- ☐ What made you decide to have the Eye Photo Test?
- ☐ Why do you think your doctor referred you for the Eye Photo Test?

Next, I have a few questions about scheduling the Eye Photo Test and getting to your appointment.

How easy was it to schedule the Eye Photo Test?

Prompts:

- ☐ How long did you wait between the time you scheduled the Eye Photo Test and when you had the appointment? (e.g. a few days, weeks or months)
- ☐ How easy was it to get to the clinic for the photo eye test? Did you drive yourself or did someone else drive you? How long did it take to get from your home to the clinic?

Now I'd like you to tell me about your experience with the Eye Photo Test itself.

Prompts:

- ☐ About how long did the Eye Photo Test take?
- ☐ Did you receive results from your Eye Photo Test?
- ☐ *[If YES]* How quickly did you get those results? Is there anything that could be changed or improved about the way you receive the results?
- ☐ *[If NO]* Did you want to receive the results from the Eye Photo Test?
- ☐ How would you like to receive your Eye Photo Test results? (e.g. letter in the mail or phone call)

How would you explain the Eye Photo Test to a friend or family member?

Prompts:

- ☐ Would you be willing to take the Eye Photo Test again? Why or why not?

For peer review only

Do you think that there is anything that could be improved about your experience with the Eye Photo Test?

Prompts:

- □ How can we make the Eye Photo Test more available to other people in your community who have diabetes?
- □ For your community, do you think $20 is a reasonable cost for the Eye Photo Test?

Do you plan to have another diabetic eye check in the future?

Prompts:

- □ *[If YES]* How soon and where might you schedule this? [verify what type of exam – Traditional Eye Exam vs Eye Photo Test]
- □ *[If NO]* Why not?

Let's look at the picture of the Traditional Eye Exam *[point to photo]***. Have you ever had this exam where an eye doctor uses eye drops to dilate and examine your eyes?**

Prompts:

- □ *[If YES]* Tell me about your experience with this exam. What was it like?

Does your insurance cover Traditional Eye Exams? How much do you normally pay to have a Traditional Eye Exam?

Let's look at the picture of the two types of eye checks again. If you had to choose between having your eyes checked using a Traditional Eye Exam or an Eye Photo Test, which would you prefer? Why?

[Jump to Part II on page 5]

For peer review only

Version II. Clinical Eye Exam

I believe you have not had the Eye Photo Test *[point to photo]***, is this correct?**

Now, let's look at the picture of the Traditional Eye Exam *[point to photo]***. Have you ever had this exam where an eye doctor uses eye drops to dilate and examine your eyes? If so, do you recall roughly the last time you had a Traditional Eye Exam?**

Next, I have a few questions about scheduling the Traditional Eye Exam and getting to your appointment.

How easy was it to schedule the Traditional Eye Exam?

Prompts:

- ☐ How long did you wait between the time you scheduled the exam and when you had the appointment? (e.g. a few days, weeks or months)
- ☐ How easy was it to get to the clinic for the Traditional Eye Exam? Did you drive yourself or did someone else drive you? How long did it take to get from your home to the clinic?

Now I'd like you to tell me about your experience with the Traditional Eye Exam itself.

Prompts:

- ☐ About how long did the exam take?
- ☐ Did you receive results from your Traditional Eye Exam?
- ☐ *[If YES]* How quickly did you get those results? Is there anything that could be changed or improved about the way you receive the results?
- ☐ *[If NO]* How would you want to receive the results from the exam?

How would you describe the Traditional Eye Exam to a friend or family member?

Prompts:

- ☐ Would you be willing to have a Traditional Eye Exam again? Why or why not?

Do you plan to have another diabetic eye check in the future?

Prompts:

- ☐ *[If YES]* How soon and where might you schedule this? [verify what type of exam – Traditional Eye Exam vs Eye Photo Test]
- ☐ *[If NO]* Why not?

Does your insurance cover Traditional Eye Exams? How much do you normally pay to have a Traditional Eye Exam?

Let's look at the picture of the two types of eye exams again. If you had to choose between having your eyes examined using a Traditional Eye Exam or an Eye Photo Test, which would you prefer? Why?

For peer review only

Part II. Wrap-up and Demographics

Is there anything else you think is important for me to know about your experience with getting diabetic eye checks?

Thanks so much for all this great information. I really appreciate your sharing your experience with me.

As I mentioned earlier, there is just one last thing—a short list of questions to get some background information. Please understand that we only want this this information so that we have an accurate picture of who is involved in our study; we know the community is diverse and we want to be sure we hear many different perspectives and hear from people with a wide range of backgrounds. We are not making any judgments or assumptions about you based on this information.

I will read the questions to you or if you prefer, you can read the questions on your own.

[Read or hand patient page 6 to complete]

For peer review only

Patient Background Information

1. How long have you had diabetes?

a) Less than 5 years
b) Between 5-10 years
c) Between 10-15 years
d) More than 15 years

2. Do you drive yourself to your eye appointments? (If no, who drives you)?

a) Yes
b) No, my ______________________________ drives me to appointments

3. During regular clinic hours, are you easily able to get your eye appointments?

a) Yes
b) No

4. What level of school have you completed?

____

5. We know that health information is often written in a way that is complicated and hard to understand. How often do you need to have someone help you when you read instructions, pamphlets, or other written material from your doctor or pharmacy?

a) Never
b) Rarely
c) Sometimes
d) Often
e) Always

For peer review only

Supplemental Appendix S2. Primary Care Provider (PCP) Interview Guide

Introduction:

Thank you so much for taking the time to talk with me. I'm going to start off by giving you an introduction and our goals for this interview.

As you know, in collaboration with Mile Bluff Medical Center (MBMC), our research team at the University of Wisconsin is studying primary care provider perspectives on diabetic eye exams.

You are being invited to participate in this interview because we are interested in learning more about your experience, as a primary care provider, with teleophthalmology and your ideas on ways to make it easier for you to use. We are also conducting separate interviews with patients to understand their experiences as well.

Participation in this study is voluntary. You may discontinue your participation at any time. There won't be any identifying information linked with your responses. Only researchers on this project will have access to the data gathered.

I will audiotape this interview so that I can take fewer notes as we talk and review our conversation later to ensure that I don't miss any information. The interview should take about 15-20 minutes. Please feel free to stop me at any time with questions or concerns.

Do you have any questions about the study before we begin?

Version I. Teleophthalmology

[NOTE: If PCP has not referred patients for teleophthalmology, skip to page 3: Version II. Clinical eye exam]

Tell me about your experience with teleophthalmology.

Prompts:

- ☐ Why did you choose to refer patients for teleophthalmology?
- ☐ What helped in that process? What didn't help in that process?
- ☐ Can you describe an instance when a referral did not happen and why?

What makes referring patients for diabetic eye exams easier or more difficult than other health maintenance screenings (e.g. mammography and colonoscopy)?

Now I'd like to ask you to walk me through your decision to refer a typical patient for teleophthalmology.

Prompts:

- ☐ Do you refer the patient? Do you have help from medical assistants or schedulers?
- ☐ How do you decide whether to refer a patient for teleophthalmology versus a traditional diabetic eye exam?
- ☐ What patient factors, such as their characteristics or preferences, influence your decision?
- ☐ What about non-patient factors that might influence your decision, such as how busy you are in clinic?
- ☐ When you see a patient with multiple health issues, at what point during a typical clinic visit might you bring up the topic of diabetic eye screening?

For peer review only

How do you explain teleophthalmology to the patient?

Prompts:

- ☐ How do patients typically respond?
- ☐ What makes you feel confident, or not, that the patient will obtain teleophthalmology screening?
- ☐ Of your patients that have had this test done, what kind of feedback do they give you?

Is there anything that might make easier for you to have your patients use teleophthalmology?

Prompts:

- ☐ Some examples may include:
  Interventions to identify patients eligible for eye screening
  Delegation of referrals to support staff
  Walk-in/flexible scheduling of eye screening
- ☐ Do you have any concerns or questions about this technology?
- ☐ Are there incentives or processes in place that encourage referring patients for this or other types of screening?

This has been very helpful. Before we conclude our conversation, is there anything else I should be asking you about teleophthalmology or diabetic eye exams that is important for me to know?

Thank you very much for your participation!

For peer review only

Version II. Clinical eye exam *[if PCP has not referred patients for teleophthalmology]*

Tell me about your experience with teleophthalmology.

Prompts:

- ☐ Do you have any concerns or questions about this technology?
- ☐ Have you considered having patients get teleophthalmology screening instead of a clinical diabetic eye exam? Why or why not?

Is there anything that might make it more likely for you to have your patients use teleophthalmology?

Prompts:

- ☐ For example:
  Interventions to identify patients eligible for eye screening
  Delegation of referrals to support staff
  Walk-in/flexible scheduling of eye screening
- ☐ Are there incentives or processes in place that encourage you or your colleagues to refer patients for this or other types of screening?

What makes referring patients for diabetic eye exams easier or more difficult than other health maintenance screenings (e.g. mammography and colonoscopy)?

Now I'd like you to walk me through the steps you or your staff take when helping patients get diabetic eye exams.

Prompts:

- ☐ Do you refer the patient? Do you have help from medical assistants or schedulers? Do patients refer themselves?
- ☐ How do you decide whether to refer a patient for a diabetic eye exam?
- ☐ What patient factors, such as their characteristics or preferences, influence your decision?
- ☐ What about non-patient factors that might influence your decision, such as how busy you are in clinic?
- ☐ When you see a patient with multiple health issues, at what point during a typical clinic visit do you bring up the topic of diabetic eye screening?
- ☐ Is there anything that could be improved about this process?

How do you explain diabetic eye exams to patients?

Prompts:

- ☐ How do patients typically respond?
- ☐ What makes you feel confident, or not, that the patient will obtain the exam?
- ☐ Of your patients that have had a diabetic eye exam, what kind of feedback do they give you?

This has been very helpful. Before we conclude our conversation, is there anything else I should be asking you about teleophthalmology or diabetic eye exams that is important for me to know?

Thank you very much for your participation!

For peer review only

COREQ (COnsolidated criteria for REporting Qualitative research) Checklist

A checklist of items that should be included in reports of qualitative research. You must report the page number in your manuscript where you consider each of the items listed in this checklist. If you have not included this information, either revise your manuscript accordingly before submitting or note N/A.

Topic	Item No.	Guide Questions/Description	Reported on Page No.
**Domain 1: Research team and reflexivity**			
Personal characteristics			
Interviewer/facilitator	1	Which author/s conducted the interview or focus group?	11
Credentials	2	What were the researcher's credentials? E.g. PhD, MD	11
Occupation	3	What was their occupation at the time of the study?	11
Gender	4	Was the researcher male or female?	11
Experience and training	5	What experience or training did the researcher have?	11
Relationship with participants			
Relationship established	6	Was a relationship established prior to study commencement?	11
Participant knowledge of the interviewer	7	What did the participants know about the researcher? e.g. personal goals, reasons for doing the research	11
Interviewer characteristics	8	What characteristics were reported about the inter viewer/facilitator? e.g. Bias, assumptions, reasons and interests in the research topic	11
**Domain 2: Study design**			
Theoretical framework			
Methodological orientation and Theory	9	What methodological orientation was stated to underpin the study? e.g. grounded theory, discourse analysis, ethnography, phenomenology, content analysis	10, 12
Participant selection			
Sampling	10	How were participants selected? e.g. purposive, convenience, consecutive, snowball	11
Method of approach	11	How were participants approached? e.g. face-to-face, telephone, mail, email	11
Sample size	12	How many participants were in the study?	11
Non-participation	13	How many people refused to participate or dropped out? Reasons?	11
Setting			
Setting of data collection	14	Where was the data collected? e.g. home, clinic, workplace	11
Presence of non-participants	15	Was anyone else present besides the participants and researchers?	11
Description of sample	16	What are the important characteristics of the sample? e.g. demographic data, date	14-15
Data collection			
Interview guide	17	Were questions, prompts, guides provided by the authors? Was it pilot tested?	10
Repeat interviews	18	Were repeat inter views carried out? If yes, how many?	13
Audio/visual recording	19	Did the research use audio or visual recording to collect the data?	11
Field notes	20	Were field notes made during and/or after the inter view or focus group?	11
Duration	21	What was the duration of the inter views or focus group?	11
Data saturation	22	Was data saturation discussed?	11
Transcripts returned	23	Were transcripts returned to participants for comment and/or	13

For peer review only

Topic	Item No.	Guide Questions/Description	Reported on Page No.
		correction?	
**Domain 3: analysis and findings**			
Data analysis			
Number of data coders	24	How many data coders coded the data?	12
Description of the coding tree	25	Did authors provide a description of the coding tree?	12
Derivation of themes	26	Were themes identified in advance or derived from the data?	12
Software	27	What software, if applicable, was used to manage the data?	12
Participant checking	28	Did participants provide feedback on the findings?	13
Reporting			
Quotations presented	29	Were participant quotations presented to illustrate the themes/findings? Was each quotation identified? e.g. participant number	16-20
Data and findings consistent	30	Was there consistency between the data presented and the findings?	15-21
Clarity of major themes	31	Were major themes clearly presented in the findings?	15-16, 21-22
Clarity of minor themes	32	Is there a description of diverse cases or discussion of minor themes?	16, 19

Developed from: Tong A, Sainsbury P, Craig J. Consolidated criteria for reporting qualitative research (COREQ): a 32-item checklist for interviews and focus groups. *International Journal for Quality in Health Care*. 2007. Volume 19, Number 6: pp. 349 – 357

Once you have completed this checklist, please save a copy and upload it as part of your submission. DO NOT include this checklist as part of the main manuscript document. It must be uploaded as a separate file.

For peer review only
